# Energy-Efficient Multi-Disjoint Path Opportunistic Node Connection Routing Protocol in Wireless Sensor Networks for Smart Grids

**DOI:** 10.3390/s19173789

**Published:** 2019-09-01

**Authors:** Junaid Anees, Hao-Chun Zhang, Sobia Baig, Bachirou Guene Lougou

**Affiliations:** 1 School of Energy Science and Engineering, Harbin Institute of Technology, Harbin 150001, China; 2 Satellite Control Facility (SCF-L) directorate, SE wing, Space and Upper Atmosphere Research Commission, Lahore 54000, Pakistan; 3 Department of Electrical and Computer Engineering, COMSATS University Islamabad (CUI), Lahore Campus, Lahore 54000, Pakistan

**Keywords:** energy-efficient wireless sensor networks, multipath opportunistic node connection, smart grids, neighborhood area network, asynchronous working–sleeping cycle strategy, energy-efficient routing protocol, opportunistic connection random graph, service differentiation in smart grids

## Abstract

The gradual increase in the maturity of sensor electronics has resulted in the increasing demand for wireless sensor networks for many industrial applications. One of the industrial platforms for efficient usage and deployment of sensor networks is smart grids. The critical network traffic in smart grids includes both delay-sensitive and delay-tolerant data for real-time and non-real-time usage. To facilitate these traffic requirements, the asynchronous working–sleeping cycle of sensor nodes can be used as an opportunity to create a node connection. Efficient use of wireless sensor network in smart grids depends on various parameters like working–sleeping cycle, energy consumption, network lifetime, routing protocol, and delay constraints. In this paper, we propose an energy-efficient multi-disjoint path opportunistic node connection routing protocol (abbreviated as EMOR) for sensor nodes deployed in neighborhood area network. EMOR utilizes residual energy, availability of sensor node’s buffer size, working–sleeping cycle of the sensor node and link quality factor to calculate optimum path connectivity after opportunistic connection random graph and spanning tree formation. The multi-disjoint path selection in EMOR based on service differentiation of real-time and non-real-time traffic leads to an improvement in packet delivery rate, network lifetime, end-end delay and total energy consumption.

## 1. Introduction

In recent years, the demand for wireless sensor networks has increased for many industrial applications such as fault diagnosis and monitoring, surveillance monitoring, industrial control systems, commodity consumption monitoring and plant automation, etc. [1]. In addition, in industrial applications, wireless sensor networks have many medical, military, civil and environmental applications as well body area network monitoring [2], civil structure monitoring [3], target tracking in battlefields [4], earthquake and flood monitoring [5], forest fire monitoring [6], etc.

The technological progression in the field of highly integrated digital sensor electronics, small scale microprocessors, low power transceivers and radio frequency devices, collectively resulted in the design of efficient wireless sensors [7]. These wireless sensor devices are responsible for sensing the change in required physical phenomena of their surrounding environment with the help of a small microprocessor, radio transceiver, a few transducers and a low capacity battery [7]. The transducers or sensors perform the specific type of sensing in the surrounding environment and pass the sensing information to a microprocessor for further processing of sensed data [7]. A wireless sensor’s radio transceiver is used to transmit and receive the sensed data to/from adjacent wireless sensors or to/from the sink node, depending upon the location of the wireless sensor in the field [7]. Due to low battery capacity, wireless sensor nodes have to adjust their sleep–awake cycle (or working–sleeping cycle) according to scenario requirements in order to maximize the network life time and minimize the overall network delay [8]. The possible existence of these miniaturized wireless sensor devices motivated researchers to emphasize the significance of wireless sensor node collaboration for data sensing, data collection and aggregation purposes which resulted in the discovery of an emerging field known as wireless sensor network (WSN). The purpose of deploying multiple wireless sensor nodes in a field is to have collaboration between these sensor nodes to achieve a shared goal with the help of sensing, data aggregation and data sharing between wireless sensor nodes [9].

The frequent transmission failures, electricity theft and congestion problems in traditional electric grids lead to the consideration that traditional electric grids are insecure and inefficient in terms of energy management. In order to eradicate these problems, we need to incorporate state-of-the-art bidirectional communication interfaces, automated control systems and distributed computing capabilities in our current grid, which will improve energy efficiency, reliability, security and agility in our electric grid [10]. Furthermore, a highly integrated next generation system is needed in which electricity service providers, distributors and prosumers are well aware of real-time energy requirements and capabilities; the system which offers high performance computing, security, scalability, reliability and security with state-of-the-art communication network infrastructure is our “smart grids” (SGs) [10,11]. For better realization of SGs, we need to distribute and gather information remotely and in a timely manner from different phases of our SGs (i.e., generation, transmission, distribution and consumption) [10,11,12]. The data acquisition from different devices deployed in the neighborhood area network (NAN) can be efficiently achieved by incorporating WSN in SGs. The upgradation of contemporary electric grids to SGs could be supported by self-governing and self-organizing features of WSN [13]. A traditional electric grid needs installation of costly and inefficient wired monitoring systems, expensive communication cables and a high-maintenance budget [10]. On the other hand, SGs with the help of wireless monitoring sensors and proper fault diagnostics, can remarkably reduce power losses and long-term maintenance budget and does not require expensive communication cables, thus enhancing the system’s reliability and efficiency [13]. 

Efficient energy utilization and network lifetime are considered as the main design parameters in the previous research conducted for WSN [8]. Additionally, the feasibility of mobility usage in WSN as demonstrated in [14] can improve the network lifetime by replacing the traditional static wireless sensor nodes with mobile wireless sensor nodes. Similarly, if the center node (or sink) is mobile, it needs more computation power in comparison to other sensor nodes [14]. The mobility and more computation power will drain out the battery of that sink node after a few rounds of sensing and aggregation, and its energy should be replenished in a timely manner. The mobility of a sink node can be controlled or randomly planned depending upon the particular scenario of the WSN and in this way, many traditional problems like hot-spot problem [14] can be avoided. With reference to [15], researchers have proposed a working–sleeping cycle strategy in which free nodes go to sleep to save their battery power and have a proper node scheduling scheme for efficient data transmission. These node scheduling schemes were categorized as synchronous and asynchronous working–sleeping cycle, which means that the network lifetime can be prolonged by changing the node scheduling schemes in accordance with the scenarios. Although it can affect the link stability between sensor nodes, it also creates an opportunistic node connection to exist between nodes due to asynchronous working–sleeping scheduling. 

According to [16,17,18,19,20], Opportunistic Routing (OR) is a paradigm for wireless networks which benefit from broadcast characteristics of a wireless medium by selecting multiple nodes as candidate forwarders, to improve network performance. In [18,19,20] a set of nodes are selected as potential forwarders and the nodes in the selected set forward the packet according to some criteria after they receive the packet. This group of nodes in OR is called a candidate set (CS). The performance of OR depends on several key factors such as OR metric, candidate selection algorithm and candidate coordination method. Boukerche et al. in [20] discussed the basic function of OR by highlighting its ability to overhear the transmitted packet and to coordinate among relaying nodes. In OR, by using a dynamic relay node to forward the packet, the transmission reliability and network throughput can be increased. Thus, the term “Opportunistic Routing” can be defined as the routing scheme in which the next best forwarder is dynamically selected with respect to OR metric, candidate selection algorithm and candidate coordinate method as in [18,19,20].

The reasons why opportunistic node connections exist in WSNs can be summarized as follows:

- WSNs are often deployed in harsh environments (smart grids NAN in our case), where wireless signals are susceptible to interference, thus causing link instability [21,22], further leading to opportunistic node connections;
- The sink node mobility usually leads to intermittent links in the network, resulting in opportunistic node connections [21];
- Due to the limited energy of the nodes, the sensor nodes adopt an asynchronous working–sleeping cycle strategy to save energy and accordingly, the adjacent nodes may not be able to communicate with each other continuously as in [22], thus bringing about opportunistic node connections.

By utilizing asynchronous working–sleeping cycle strategy, multiple nodes in WSNs can apply the concept of OR by overhearing their working neighbor’s transmission. A set of potential forwarders can be created using opportunistic connection random graph (OCRG) and formation of a spanning tree can be used to demonstrate that the candidate nodes in OR will forward the packet according to some criteria (i.e., optimal link and path connectivity calculations in our case).

However, the sensed data being generated as a result of deploying a WSN in a NAN of SGs might have different attributes like delay tolerance and delay sensitivity [13]. For example, the monitoring data (e.g., power load) generated by a sensor network which is part of a SG can always be delay sensitive and should be transmitted to the data processing (or sink) node within certain time limits thereby increasing the bandwidth requirements, whereas the control data (e.g., changing power load) generated by the sensor network in SGs can often be considered as delay tolerant data and is not required to be received by the data processing (or sink) node immediately [13]. Keeping in view the system requirements of SGs, parameters like energy consumption, mobility, network lifetime, delay and bandwidth can be treated as performance metrics for effective use of the WSN in SGs. 

**Figure 1:**
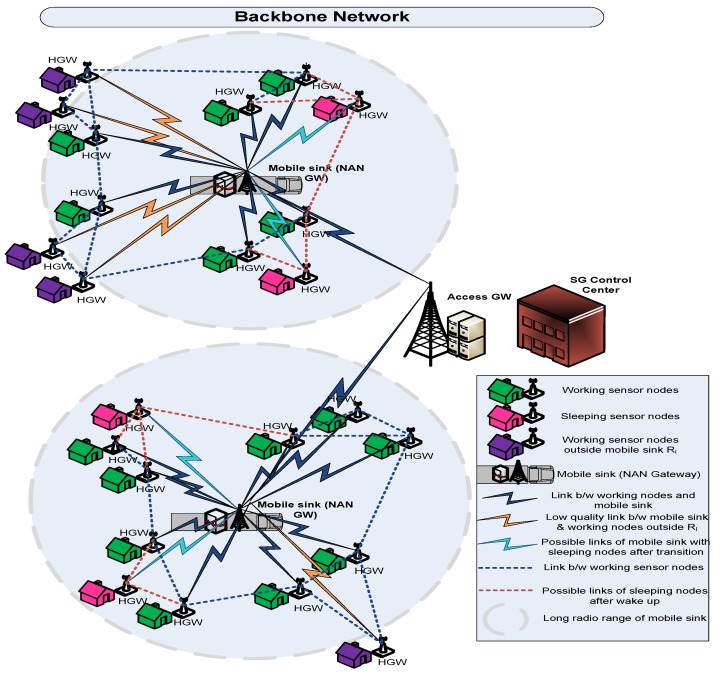
Illustration of wireless sensor network (WSN) adopting working–sleeping cycle strategy with opportunistic node connections in neighborhood area network (NAN).

Many routing techniques specifically designed for WSNs have been proposed in [23,24,25]. Likewise, the multipath routing protocols according to [26] have been proposed for WSNs. These multipath routing protocols use multiple paths for data delivery, and thus improve the network reliability and robustness. In this paper, we propose an energy efficient and multipath opportunistic node connection routing protocol for WSNs in NANs to achieve load balancing through splitting up traffic in terms of real-time and non-real-time across multi-disjoint paths and energy consumption balance through asynchronous working–sleeping cycle of sensor nodes. Figure 1 demonstrates the wireless sensor networks deployed in the NAN of SGs in which our NAN gateway is actually the mobile sink. The opportunistic node connections between mobile sink sensor nodes and between adjacent sensor nodes can be clearly seen in Figure 1.

As a reflection of opportunistic node connectivity caused by asynchronous working–sleeping cycle strategy, OCRG is constructed in which a probe message forwarding mechanism is used for each node to forward the information to the mobile sink node [27]. Additionally, EMOR utilizes the residual energy, availability of the node’s buffer size, working–sleeping cycle of sensor nodes and link quality factor to calculate the optimal link and path connectivity for both real-time and non-real-time traffic and assigns multi-disjoint paths to them accordingly.

The rest of the paper is organized as follows: Section 2 describes the related research work conducted for WSN routing protocols in SGs and various other applications. System modeling is presented in Section 3. EMOR protocol design for WSNs in NAN is presented in Section 4. Performance evaluation and simulation results can be depicted from Section 5. Finally, Section 6 and Section 7 briefly discuss the paper and provide some future research directions.

## 2. Related Research Work

Keeping in view the need for a more efficient and robust electric grid, a conceptual framework was proposed by the National Institute of Standards and Technology (NIST) in SGs Interoperability Standards Roadmap [28,29]. According to this conceptual framework, there is a need to have an inter domain and intra domain communication between key building blocks like operations, markets, service providers, generation, transmission, distribution and consumer [28,29]. The NIST framework states that SGs should support the information flow and electrical flow between all key building blocks. Information flow includes data acquisition, data processing and data dissemination between the desired energy subsystems of SGs, while the electrical flow deals with the generation, transmission and distribution of energy [29]. The main objective of information flow is to monitor and control the energy whereas the electrical flow is responsible for power delivery, demand and asset optimization, etc. [29].

In [30,31,32], several key technologies have been identified by the National Energy Technology Laboratory (NETL) for SGs:

(1) Full-duplex and high-speed communication infrastructure for the information exchange between different SG entities.
(2) State-of-the-art sensing network infrastructure which will be responsible for the measurement and relaying of physical data. This sensor network can be used to prevent power theft, improve demand response, etc.
(3) Fabrication of components related to power electronics, superconductivity and energy storage should be needed based on the contemporary research being conducted for SGs.
(4) Seamless and real-time decision making at the prosumer end should be possible by improving the prosumer interface with operations, markets and service providers.

The general overview of SGs concept is presented in [11,12]. The communication architecture and application needs of SGs are explained in detail in [30]. Moreover, the mechanisms for data collection and SGs sensing network is surveyed in [13] and strategies for improving information flows in SGs is mentioned in [33]. Technologies like high speed communication network architecture for SGs have been studied in [30]. Due to the decentralized and lightweight architecture of WSNs, it can be efficiently used in SGs and micro-grids [13]. Erol-Kantarci et al. in [33] presented that WSNs can be used in various SG applications like generation, transmission, distribution and consumption as the WSN can deliver the information needed by the intelligent algorithms running in the control center of SGs. An overview of opportunities and challenges of WSNs in SGs is presented in [34]. Likewise, different researchers have focused on proposing different routing protocols for WSNs based on parameters like network lifetime, delay, bandwidth, packet to delivery ratio and node mobility [23,24,25,26]. As mentioned in a detailed survey for energy efficient and energy balanced routing protocols for a WSN [35], most of these protocols emphasize on improving energy consumption during network transmission activities but very few researches can be found about improvement in energy consumption with node mobility. 

In a typical WSN topology, all nodes including the sink nodes are static. This leads to a situation in which the adjacent or neighboring nodes of the sink node deplete their limited battery powers as they have to participate in managing more traffic loads for forwarding the sensing data to the sink node as compared to the nodes which are far from the sink node. This typical scenario is known as hot-spot problem and adversely affects the network lifetime in any WSN application [14,15]. For this particular reason, the node mobility concept is introduced by many researchers, which exploits the movement of the sink node in the WSN to overcome the hot-spot problem [36,37]. As the location of the neighboring nodes around the sink node are changed due to movement of the sink node, the probability of a hot-spot problem is reduced and network lifetime is improved due to a more even distribution of energy consumption in the surrounding nodes [37]. Keeping in view the node mobility, researchers have worked on the path formation for mobile sinks in [37,38,39] based on the predefined, controlled and random path selection procedures. In [40], Alghamdi et al. proposed a new routing aware algorithm to detect malicious nodes in a concealed data aggregation for WSNs to highlight the significance of data aggregation in WSNs by reducing communication overhead of sensor nodes.

All these previous researches were formulated to equalize the energy consumption of nodes, but they are not able to prolong the network lifetime. Thus, an emerging concept “utilization of working–sleeping cycle of nodes” was introduced to improve the energy consumption of nodes and overall network lifetime [22]. The working–sleeping cycle can be segmented into two categories—synchronous and asynchronous working–sleeping cycles. Authors in [41,42] revealed that synchronous working–sleeping cycle could help in achieving improvement in energy consumption. However, the synchronization problem needed significant contributions. Thus in [43], Ng et al. proposed an energy efficient synchronization algorithm in which adaptive adjustment of the traffic and wakeup period could improve energy consumption through counter-based and exponential-smoothing algorithms. In addition to it, some researchers also explored asynchronous working–sleeping cycle in which all nodes have independent working–sleeping schedules depending on the network connectivity requirements in terms of traffic coverage area [16,17]. Mukherjee et al. also proposed an asynchronous working–sleeping technique while focusing on network coverage by maintaining a minimum number of awake nodes [44]. As a result of working–sleeping cycle strategy, opportunistic node connections will be established between sensor nodes and the mobile sink which can further lead to link instability. Therefore, we need a random graph theory to model these kinds of opportunistic node connections in the WSN. Mostafaei et al. proposed reliable routing with distributed learning automaton (RRDLA) algorithm, which considers dynamics of links in finding a path from a source to a destination by considering Quality of Service (QoS) constraints such as end-to-end reliability and delay [45]. Ben Fradj et al. in [46] presented a new opportunistic routing protocol called energy-efficient opportunistic routing protocol using a new forward list (EEOR-FL) aiming to balance energy consumption and maximize the network lifetime by calculating the list of candidates. Sadatpour et al. proposed a new collision-aware opportunistic routing protocol abbreviated as SCAOR for highways by utilizing cluster-based scheduling algorithms [47]. 

Kenniche et al. in [48] indicated that WSN modeling in the case of working–sleeping cycle strategy and opportunistic node connections can be modeled using a random geometric graph in which a set of vertices represent sensor nodes and a set of edges represent links between those vertices or sensor nodes. In reference [49], Norman et al. proposed a novel random graph modeling for heterogeneous sensor networks based on different transmission ranges and a new routing metric. Referring to [50], in which Ren et al. focused on the challenges and weaknesses of using random graph theory for modeling WSN, they thus proposed a new weighted topology model for WSN based on the random geometric theory. In [51], Fu et al. developed an optimal policy for connection between source and destination nodes in random WSN topology using random graph. For this policy to be implemented correctly, an energy efficient probe message forwarding mechanism was proposed. After analyzing the source information in the received probe message, the mobile sink calculates the link connectivity between any two adjacent nodes after determining their neighbor relationships. Thus, an opportunistic connection random graph can be constructed. 

As SGs require real-time (delay sensitive) and non-real-time (delay tolerant) traffic being routed through the WSN while minimizing the energy consumption and increasing the network lifetime, we need to utilize the WSN routing protocol which can offer energy efficiency and multi-disjoint path routing. Keeping in view these SGs requirements, Dulman et al. in [52] discussed the trade-offs between traffic overhead and reliability in multi-path routing for WSN. In [35], the authors discussed the taxonomy of cluster-based routing protocols for WSN, with respect to energy efficient and energy balance routing protocols. Ben-Othman et al. discussed the energy efficient and QoS based routing protocol in [53]. Mostafaei et al. in [54] investigated the problem of self-protection in WSNs and devised the Self Protection Learning Automaton (SPLA) algorithm in which sensing graph of the network plays the main role in finding the minimum number of nodes to protect the nodes. The proposed solution takes advantage of irregular cellular learning automaton (ICLA) to properly schedule the sensors into either an active or idle state. In [53], researchers proposed an energy efficient and QoS-based multi-path routing protocol with disjoint paths for real-time and non-real-time traffic management. In [55], Liang et al. presented a novel routing optimization technique based on improvement in Low Energy Adaptive Clustering Hierarchy (LEACH) in WSNs. According to this novel routing optimization technique, energy efficiency per unit node per round can be achieved and network lifetime can be prolonged. In our proposal, we utilized certain ideas from the previous routing protocols in WSN and proposed a detailed solution for optimally tackling the problems like energy consumption and network lifetime enhancement through multi-disjoint path opportunistic node modeling using random graph theory for delay-tolerant and delay-sensitive data in SGs NAN.

## 3. System Modeling

**Figure 2:**
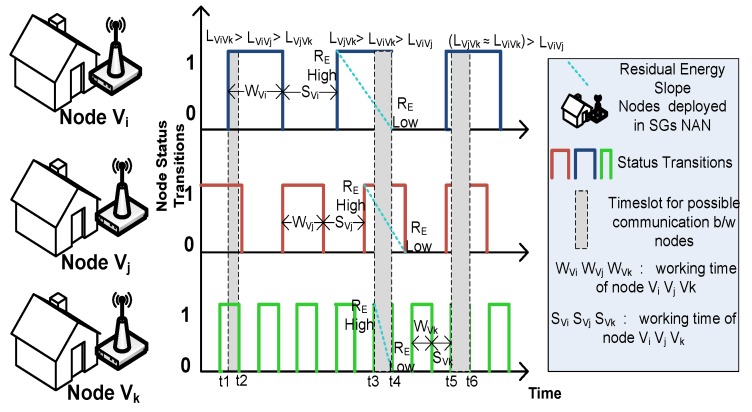
Illustration of link connectivity based on asynchronous working–sleeping cycle strategy and residual energy of nodes deployed in NAN. SGs—smart grids.

In this paper, we assumed that all sensor nodes are being deployed in the NAN randomly and independently and the mobile sink is considered as the NAN gateway for collection of monitoring and control data from the sensor nodes. Also, we assumed that all sensor nodes follow asynchronous working–sleeping cycle strategy with their working time as *W_V_* and their sleeping time as *S_V_*. The residual energy of the sensor nodes is based on the simplified energy consumption model [18]. Figure 2 depicts the asynchronous working–sleeping cycle strategy of nodes along with their residual energy slope. Both of these features are used to visualize the possible link connectivity between adjacent sensor nodes, *V_i_*, *V_j_*, and *V_k_*, which are within their radio range r. With reference to [18], the energy consumed during transmission and reception for adjacent nodes *V_i_* and *V_j_* is given by:

(1) $$E_{T}(V_{i},V_{j})=(E+\varepsilon_{amp}d_{\left(V_{i},V_{j}\right)}^{2})B$$

(2) $$E_{R}(V_{j})=EB$$

where *E* is the basic energy consumed during transmission and reception per bit, *B* is the number of bits transmitted or received, $\varepsilon_{amp}$ is the energy consumed by transmission amplifier, $d_{\left(V_{i},V_{j}\right)}$ is the distance between node *V_i_* and *V_j_*, *E_T_* and *E_R_* are the energy consumed by nodes for transmission and reception, respectively. Figure 2 shows that the link connectivity between adjacent nodes depends on asynchronous working–sleeping cycle as well as residual energy of nodes. When the node is in working mode, its residual energy starts depleting and follows the residual energy slope. The residual energy at the start of the working mode is high and is low at the end of the working mode. So, the opportunistic link connection if established at the start of the working mode will lead to higher link connectivity value as compared to the connection established at the center or end of the working mode. 

## 4. EMOR Protocol Design for WSN in NAN

The following five phases reflect the proposed design of EMOR for WSN deployed in NAN. 

### 4.1. Initialization Phase

It is assumed that the mobile sink node can start the data collection anywhere and anytime in the network by broadcasting a message which contains the sink ID (SID) and data collection duration. Upon receiving the message from the mobile sink, the sensor nodes within long radio range *R_l_* obtain the data collection duration the sink and start calculating their working schedule (based on the working–sleeping cycle) and status transition frequency (i.e., switching from working to sleeping and vice versa), within this particular data collection duration. In case the sensor node is not working (sleeping state), then it can participate in the next data collection period when the sink node broadcasts the same message again to facilitate the sleeping sensor nodes and acquisition of probe messages from the new sensor nodes. Here, we have assumed that the relative signal strength indication (RSSI) parameter can be used to estimate the distance between the mobile sink and sensor nodes. In order to minimize the energy consumption, we employed the expected optimal hops (EOH) approach. EOH can be defined as the number of hops needed to forward a probe message from any node to the mobile sink node with minimum energy consumption [22]. According to [22], EOH can be expressed as:

(3) $$EOH_{V_{i}}=\sqrt{\frac{\varepsilon_{amp}}{2E}}d_{\left(V_{i},V_{\sin k}\right)}$$

where *E* is the basic energy consumed during transmission and reception per bit, $\varepsilon_{amp}$ is the energy consumed by the transmission amplifier, $d_{\left(V_{i},V_{\sin k}\right)}$ is the distance between node *V_i_* and *V_SINK_* and $EOH_{V_{i}}$ is the expected optimal hops of node *V_i_* needed to select proper forwarders to the mobile sink with minimum energy consumed.

### 4.2. Probe Message Forwarding Mechanism in Data Collection Scope

All sensor nodes within long radio range *R_l_* which received the broadcast message from the mobile sink, need to send a data set comprising of information such as source ID, status transition frequency, working–sleeping schedule and all neighbor IDs to the mobile sink node during the data collection period. The format of probe message being forwarded to the mobile sink can be seen from Table 1.

**Figure 3:**
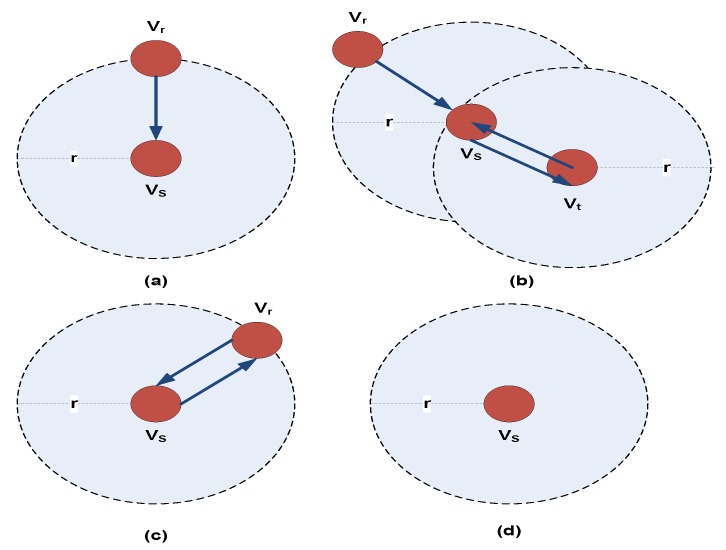
Probe message receiving and forwarding mechanism in EMOR. (**a**) *Vs* receives probe message from *Vr*; (**b**) *Vs* forwards the received probe message to working neighbor *V_t_*; (**c**) *V_t_* has only one neighbor *Vs* to forward the probe message; (**d**) *Vs* has no neighbor.

The length of the probe message is 120 bits in which the source ID occupies 10 bits, working–sleeping schedule occupies 15 bits, status transition frequency and neighbor IDs have 10 bits each, sink ID has 10 bits, forwarders ID has 60 bits and EOH contains 5 bits. The “Source ID” represents the ID of the node sending the message to the mobile sink. The “Working–Sleeping Schedule” represents the information about whole working time and whole sleeping time of the respective node. “Status Transition Frequency” provides information about the transitions from working mode to sleeping mode and vice versa of the sensor node. “Neighbor IDs” includes the ID list of neighboring nodes to the source node. If there is no neighbor or all neighboring nodes are in sleeping mode, the “Neighbor IDs” field remains empty. Moreover, the “Sink ID” is related to mobile sink’s SID and is used to indicate that the probe message being forwarded from the sensor node is specifically targeted for the desired sink node in case of multiple mobile sinks present in the network. “Forwarders ID” stores the IDs of nodes which have already forwarded this probe message to the mobile sink. This field is updated when a new node forwards this probe message to the mobile sink and adds its ID in the forwarders ID list. Likewise, “EOH” field stores the value of expected optimal hops needed for the source ID message to reach the sink node. As this probe message can be generated by any sensor node within the radio range *R_l_* of the mobile sink, so it’s quite probable that different working–sleeping schedules and status transition frequencies of sensor nodes can lead to inefficient probe message forwarding. Therefore, only the nodes that have received the probe message can participate in this forwarding mechanism. For example, for an intermediate node *V_S_* receiving the probe message from one of its neighbors *V_r_* as shown in Figure 3, the first step would be to check its own ID in the “Forwarders ID” field. If the probe message was not forwarded by this node *V_S_*, it will add its ID to “Forwarders ID” field, determine the total number of forwarders (TNF) for this probe message and then calculate (EOH-TNF). Now it will check the “Neighbor IDs” field and see which neighbor’s EOH is closest to (EOH-TNF) as an optimal forwarder.

Since the asynchronous working–sleeping cycle strategy is used in sensor nodes, if any sensor node *V_S_* receives the probe message from its surrounding node *V_r_*, it will forward the probe message to one of its neighbors only if that neighbor node is in working mode. Likewise, it will not forward the probe message if there is no neighboring node or the neighbor is in sleeping mode. The probe message receiving and forwarding mechanism based on asynchronous working–sleeping cycle of nodes is depicted in Figure 3. Algorithm 1 provides the details about probe message forwarding mechanism.


**Algorithm 1: Initialization and Probe Message Forwarding Mechanism in EMOR**

**Input:**
1.Mobile sink broadcasting SID and data collection duration to all nodes within *R_l_*2.Sensor nodes within *R_l_* receive the data collection message
**Output:**
3.Select neighbor which is optimal forwarder
**Begin:**
4.Calculate the working–sleeping cycle and status transition frequencies5.Estimate the distance between sensor node and mobile sink using RSSI6.Calculate EOH based on distance estimation $d_{\left(V_{i},V_{\sin k}\right)}$7.Determine the optimal forwarders based on the following criteria:8.When an intermediate working node *V_s_* receives the mobile sink message9.***if*** (*V_s_* has not forwarded the probe message to any of its neighbors), ***then***10.     Add *V_s_* source ID in the forwarders ID list and calculate total number of forwarders (TNF)11.  Compute $EOH_{V_{S}}-TNF$12.  ***if*** (*V_s_* has working neighbors), ***then***13.    Check EOH of all of its working neighboring nodes14.    ***if*** $\left(EOH_{NE}\approx EOH_{V_{S}}-TNF\right)$, ***then***15.      Select the neighbor as an optimal forwarder16.    ***end if***17.  ***endif***18.***else if*** (*V_s_* has already forwarded probe message to neighbor node *V_t_* but receives it again), ***then***19.  Explore the working neighbors other than *V_t_* that have not forwarded the message yet20.  Remove the recently added source IDs of *V_s_*, *V_t_* from forwarders ID list21.  Add *V_s_* source ID in the forwarders ID list and calculate total number of forwarders (TNF)22.   Compute $EOH_{V_{S}}-TNF$23.  ***if*** (*V_s_* has working neighbors other than *V_t_*), ***then***24.    Check EOH of working neighboring nodes other than *V_t_*25.    ***if*** $\left(EOH_{NE}\approx EOH_{V_{S}}-TNF\right)$, ***then***26.      Select the neighbor as an optimal forwarder27.    ***end if***28.  ***end if***29.
***endif***
30.***if*** (all *V_s_* neighbors are sleeping), ***then***31.  Stop forwarding the probe message32.  Wait for the next data collection by sink node.33.
***end if***



### 4.3. Construction of Opportunistic Connection Random Graph

The construction of OCRG is dependent on the asynchronous working–sleeping cycle of sensor nodes and additional source information received within probe message forwarding during the initialization phase. In order to construct an OCRG, we assumed that the mobile sink is always in working mode and any sensor node within the short radio range *R_S_* of the mobile sink can communicate with the mobile sink at any time. Furthermore, we proposed a data set *D(S,N_s_,W/S,F_ST_,R_E_,B_S_,S/N)* consisting of source (*S*) and its neighboring nodes (*N_S_*) information, working–sleeping cycle schedule (*W/S*), status transition frequencies (*F_ST_*) for every sensor node, residual energy of every sensor node after every round of communication (*R_E_*, buffer size (*B_S_)* and signal-to-noise ratio *(S/N)*) of every sensor node with its adjacent nodes. Using this data set *D(S,N_s_,W/S,F_ST_,R_E_,B_S_,S/N)*, we analyzed the opportunistic connection between any adjacent sensor nodes which are in working mode. Additionally, for EMOR, the link connectivity between adjacent nodes is dependent on asynchronous working–sleeping cycle *W/S*, status transition frequencies *F_ST_* of the adjacent nodes, residual energies of the adjacent nodes *R_E_*, remaining buffer size of the adjacent nodes to cache the sensory data and link quality factor between adjacent nodes in terms of signal-to-noise ratio *S/N*. More status transitions of a node will lead to an improvement in its link connectivity with the adjacent node as the probability of establishing a link connection will increase. Keeping in view the data set *D(S,N_s_,W/S,F_ST_,R_E_,B_S_,S/N)*, the time-frequency parameter *TF_ViVj_* of link connectivity *L_ViVj_* can be calculated as:

(4) $$TF_{ViVj}\;=\left(\frac{F_{STVi}}{F_{STmax}}\times \frac{W_{Vi}}{T_{CP}}\right)\left(\frac{F_{STVj}}{F_{STmax}}\times \frac{W_{Vj}}{T_{CP}}\right)$$

(5) $$TF_{ViV_{SINK}}\;=\left(\frac{F_{STV_{i}}}{F_{STmax}}\times \frac{W_{V_{i}}}{T_{CP}}\right)\left(W_{V_{SINK}}\right)$$

where *W_Vi_ and W_Vj_* are the whole working time of the adjacent nodes *V_i_* and *V_j_*, *T_CP_* is the data collection period during probe message forwarding mechanism, *F_STVi_* and *F_STVj_* are the status transition frequencies of adjacent nodes *V_i_* and *V_j_*, *TF_ViVj_* is the time-frequency parameter of link connectivity *L_ViVj_*, and *F_STmax_* is the max status transition frequency value obtained during *T_CP_* which is used here for normalization of the sensor node’s *F_ST_*. The mobile sink is always in working mode, so *W_SINK_* = 1 and Equation (5) will be become,

(6) $$TF_{ViV_{SINK}}\;=\left(\frac{F_{STV_{i}}}{F_{STmax}}\times \frac{W_{V_{i}}}{T_{CP}}\right)$$

**Figure 4:**
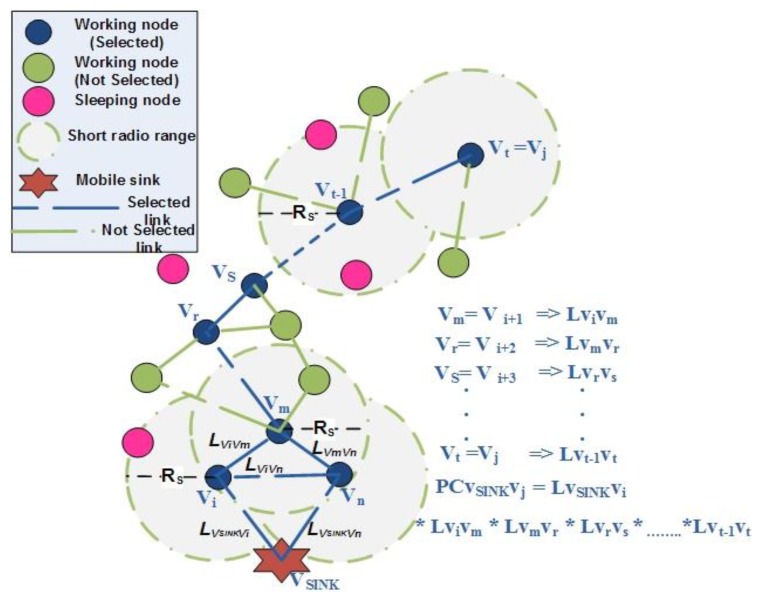
Representation of opportunistic connection random graph (OCRG) based on the link connectivity.

Hence, the time-frequency parameter $TF_{ViV_{SINK}}$ only depends upon the whole working time and status transition frequency of the sensor nodes. With time-frequency parameter TF, we have to calculate the residual energy, remaining buffer size and link quality factor between adjacent nodes. In order to determine the next best hop, we assume that there are N nodes deployed in the NAN, so our link connectivity function *L_ViVj_* in terms of *TF, R_E_, B_S_, S/N* will be:

(7) $$L_{ViVj}=\underset{Vj\in N}{\max}\{\alpha TF_{ViVj}+\beta R_{E,Vj}+\gamma B_{S,Vj}+\sigma S/N_{ViVj}\}$$

(8) $$L_{V_{SINK}V_{i}}=\underset{V_{i}\in N}{\max}\{\alpha TF_{V_{SINK}V_{i}}+\beta R_{E,V_{i}}+\gamma B_{S,V_{i}}+\sigma S/N_{V_{SINK}V_{i}}\}$$

where $R_{E,V_{j}},R_{E,V_{i}}$ are the contemporary residual energies of node *V_j_* and *V_i_*, $B_{S,V_{j}},B_{S,V_{i}}$ are the available buffer size of nodes *V_j_* and *V_i_*, $S/N_{V_{i}V_{j}},S/N_{V_{SINK}V_{i}}$ are the link quality factors in terms of signal-to-noise ratio between *V_i_* and *V_j_* and *V_SINK_* and *V_i,_* respectively. *TF_ViVj_* is the time-frequency parameter of link connectivity *L_ViVj_*, α,β,γ,σ are the appropriate weights assigned to time-frequency parameter, residual energy, buffer size and link quality factor, respectively. Also, we have considered the residual energy and buffer size of node *V_j_* only in Equation (7) because node *V_j_* consumes energy and buffer capacity for both data reception and transmission in accordance with a simplified energy model [18]. Moreover, illustration of opportunistic connection random graph to understand the link and path connectivity based on asynchronous working–sleeping cycle of adjacent nodes can be seen in Figure 4. 

### 4.4. Optimal Paths Calculation and Spanning Tree Design

After the construction of OCRG, we need to find the optimal path in EMOR for our sensor node’s data to reach the sink node successfully. In order to establish the optimal path, we need to design a spanning tree algorithm which will help us in determining the maximum value of path connectivity from any sensor node to mobile sink node. Using this algorithm, we will select the optimal multi-disjoint paths for real-time and non-real-time traffic towards mobile sink (NAN gateway) in Section 4.5. 

Path connectivity (PC) depends on the link connectivity of adjacent sensor nodes and is calculated as the product of individual link connectivity values between sensor nodes along the path towards the sink node. The reliability of the data delivery depends on the link connectivity between adjacent sensor nodes and optimal path selection. PC for a path (*V_i_*, *V_i+t_*, t) according to Figure 4 can be formulated in Equation (9) as:

(9) $$PC_{V_{i}V_{j}}=PC_{ViVi+t}\;=\prod_{k=0}^{t-1}L_{V_{i+k},V_{i+(k+1)}}\;$$

Similarly, the PC for neighboring nodes *V_i_* of mobile sink node *V_SINK_* will be:

(10) $$PC_{V_{SINK}V_{i}}\;=L_{V_{SINK},V_{i}}$$

During the construction of the spanning tree, it is pertinent to mention that the nodes whose optimal path are formed already, since they were the neighbors of mobile sink, can help the mobile sink node in getting the optimal paths of intermediate and far-end nodes. Equations (8), (9) and (10) help us in determining the PC of node *V_j_* to mobile sink. The PC from mobile sink node to an unknown sensor node *V_j_* depends on 2 factors: (i) PC between mobile sink and its neighboring nodes and (ii) link connectivity between mobile sink’s neighboring node *V_i_* and unknown sensor node *V_j_*.

(11) $$PC_{V_{SINK}V_{j}}\;=PC_{V_{SINK}Vi}\times \prod_{k=0}^{t-1}L_{V_{i+k},V_{i+(k+1)}}\;$$

(12) $$PATH(V_{SINK},V_{j},*)=\{V_{SINK},V_{j},t+1\}=\{V_{SINK},V_{i},\ldots V_{i+t-1},V_{j}\}$$

where $PC_{V_{SINK}Vi}$ is the path connectivity from the mobile sink to neighboring node *V_i_*. Here we have assumed that *V_j_ = V_i+t_* for which *t* is the number of hops on the path from *V_i_* to *V_j_* and it satisfies the criteria 1≤t≤N−1. In every round of communication (iteration), the mobile sink acquires the updated path connectivity values of intermediate and far-end sensor nodes deployed in the NAN, compares it with the previous PC values, and selects the max PC value. The same process is repeated until mobile sink node receives the updated path and max PC information of all the nodes present in the network.

(13) $$PC_{{}_{V_{SINK}V_{j}}}^{updated}=\max\{PC_{V_{SINK}V_{j}},PC_{V_{SINK}V_{i}}\times \prod_{k=0}^{t-1}L_{{}_{V_{i+k},V_{i+(k+1)}}}^{updated}\}$$

(14) $$PATH(V_{SINK},V_{j},*)=\{V_{SINK},V_{j},t+1_{updated}\}=\{V_{SINK},V_{i},\ldots V_{i+t-1(updated)},V_{j}\}$$

This spanning tree formation is useful in reducing the overall delay in the network as if the mobile sink needed to explore all the possible paths before selecting the optimal path and determining which one has maximum PC value would have resulted in more delay and a less efficient spanning tree design.

**Figure 5:**
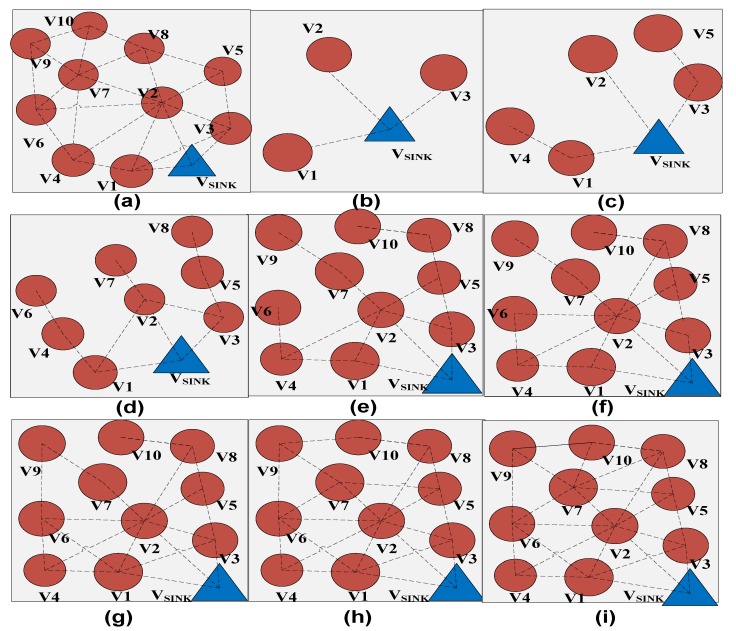
Formation of spanning tree based on OCRG. (**a**) OCRG with V_SINK_ and 10 sensor nodes; (**b**) initialization and path connectivity of immediate neighbors of V_SINK_; (**c**) path connectivity of V_4_ and V_5_; (**d**) path connectivity of V_6_, V_7_ and V_8_; (**e**) path connectivity of V_9_ and V_10_; (**f**) alternate paths for immediate nodes of V_SINK_; (**g**) alternate paths for intermediate and far end nodes; (**i**) spanning tree completed.

The formation of a spanning tree and optimal path calculation after construction of OCRG can be seen in Figure 5 and Table 2. It can be depicted from Figure 5b that after construction of OCRG, the mobile sink starts the initialization phase by connecting to its immediate neighbors. Figure 5c shows the path connectivity between the mobile sink and neighbors of the neighbors of mobile sink (i.e., node V_4_ and V_5_). The process of spanning tree formation continues from Figure 5d–i based on the optimal path connectivity rule in Algorithm 2. According to the optimal path connectivity rule, we have to choose the maximum values between the current path connectivity value and updated path connectivity value. The optimal path connectivity values for Figure 5 are given in Table 2. The initialization step of Table 2 is synchronized with Figure 5b where only immediate neighbors are connected to the mobile sink. From steps 1–7, the optimal path connectivity rule is followed to reduce the overall delay in the network.
**Algorithm 2: Optimal Paths Calculation and Spanning Tree Design for Non-Distinguishable Service****Input:**1.Formation of data set *D(S,N_s_,W/S,F_ST_,R_E_,B_S_,S/N)*2.Construction of opportunistic connection random graph (OCRG)**Output:**3.Optimal path connectivity and spanning tree formation**Begin:**4.Determine link connectivity *L_ViVj_* in terms of *TF, R_E_,B_S_, S/N* of adjacent nodes5.$L_{ViVj}=\underset{Vj\in N}{\max}\{\alpha TF_{ViVj}+\beta R_{E,Vj}+\gamma B_{S,Vj}+\sigma S/N_{ViVj}\}$6.Set the path connectivity of *V_SINK_*, i.e., $PC_{V_{SINK}V_{SINK}}\;=1;$7.Determine the path connectivity for neighboring nodes *V_i_* of mobile sink node *V_SINK_*8.$PC_{V_{SINK}V_{i}}\;=L_{V_{SINK},V_{i}}$9.***for*** each awake node *V_j_* in network, ***do*** //subroutine_wakeup10.  ***if*** (*V_j_* is directly connected to neighboring node *V_i_* of mobile sink node *V_SINK_*), ***then***11.     $PC_{V_{SINK}V_{j}}\;=L_{V_{SINK},V_{i}}\times L_{V_{i},V_{j}}$12.     $PATH(V_{SINK},V_{j},*)=\{V_{SINK},V_{j},2\}=\{V_{SINK},V_{i},V_{j}\}$13.  ***elseif*** (*V_j_* is not directly connected to neighboring nodes *V_i_* of mobile sink node *V_SINK_*), ***then***14.     $PC_{V_{SINK}V_{j}}\;=PC_{V_{SINK}Vi}\times \prod_{k=0}^{t-1}L_{V_{i+k},V_{i+(k+1)}}\;\mathrm{where}V_{i+t}=V_{j}$15.     $PATH(V_{SINK},V_{j},*)=\{V_{SINK},V_{j},t+1\}=\{V_{SINK},V_{i},\ldots V_{i+t-1},V_{j}\}$16.  ***else***  $PC_{V_{SINK}V_{j}}\;=0;$17.     $PATH(V_{SINK},V_{j},*)=\emptyset ;$18.  ***end if***19.***end for***20.***for*** each sleeping node *V_j_* in network, ***do***21.  ***if*** (*V_j_* wakes up); it should send the probe message to mobile sink with updated information22.     *V_SINK_* should reconstruct OCRG in the next iteration23.     subroutine_wakeup: go to 9 //execute steps 9–2024.  ***end if***25.***end for***26.***while*** (PC from *V_SINK_* to entire network is not yet established)27.  Select the optimal path using following expression28.  $PC_{{}_{V_{SINK}V_{j}}}^{updated}=\max\{PC_{V_{SINK}V_{j}},PC_{V_{SINK}V_{i}}\times \prod_{k=0}^{t-1}L_{{}_{V_{i+k},V_{i+(k+1)}}}^{updated}\}$29.  Update the path from *V_SINK_* to *V_j_*30.  $PATH(V_{SINK},V_{j},t+1)=\{V_{SINK},V_{j},t+1_{\left(updated\right)}\}=\{V_{SINK},V_{i},\ldots V_{i+t-1(updated)},V_{j}\}$31.***end while***

After the formation of spanning tree, the mobile sink should broadcast it to the sensor nodes within long radio range *R_l_* in every round of communication. The sensor nodes send the sensing data to mobile sink using this spanning tree information. If a sensor node does not find itself on the spanning tree, it can wait for the next broadcast message from the mobile sink during data collection period and resend its probe message to mobile sink. Upon receiving this probe message, the mobile sink will reconstruct OCRG, re-formulate the spanning tree by re-calculating the optimal path from itself to that node. 

### 4.5. Optimal Multi-Disjoint Path Selection for Real-Time and Non-Real-Time Traffic

We know that the link connectivity function $L_{V_{i}V_{j}}$ depends on several factors such as time-frequency parameter, residual energy of each neighboring sensor node, remaining buffer size and link quality factor in terms of signal-to-noise ratio. Based on the link connectivity function, we select our next best hop to create an optimal link between sensor node and one of its neighboring nodes, which further results in the optimal path connectivity towards mobile sink node. But this approach just includes the same optimal path for all kinds of data delivery, which will not suit delay-sensitive and delay-tolerant traffic requirements in SGs. Keeping in view the SGs control and monitoring data requirements in NAN, we need to split up our real-time and non-real-time data in such a way that instant priority and an optimal multi-path connectivity should be provided to real-time traffic, whereas for non-real-time traffic, secondary priority should be assigned, and an alternate multi-disjoint path connectivity should be provided. 

Therefore, we need to find the next most preferred neighboring node (second best hop) for an alternative multi-disjoint path connectivity of non-real-time traffic. In this way, we can have two-path connectivity:

(a) Primary multi-path connectivity for real-time traffic based on first best hop decision criteria in link connectivity function
(b) Alternate multi-path connectivity for non-real-time based on second best hop decision criteria in link connectivity function
  (15) $$\left\{ \begin{array}{l} L_{ViVj}^{RT}=\underset{Vj\in N}{\max}\{\alpha TF_{ViVj}+\beta R_{E,Vj}+\gamma B_{S,Vj}+\sigma {\left(S/N\right)}_{ViVj}\}\ldots \ldots \ldots H1:real-time\\ L_{ViVj}^{NT}=\underset{Vj\in N}{2nd\max}\{\alpha TF_{ViVj}+\beta R_{E,Vj}+\gamma B_{S,Vj}+\sigma {\left(S/N\right)}_{ViVj}\}\ldots H2:non-real-time \end{array}\right.$$
  (16) $$\left\{ \begin{array}{l} PC_{Vi,Vi+t}^{RT}\;=L_{{}_{V_{i},V_{i+1}}}^{RT}\times \prod_{k=1}^{t-2}L_{V_{i+k}V_{i+(k+1)}}^{RT}\;\times L_{V_{i+t-1}V_{i+t}}^{RT}\ldots \ldots H1:real-time\\ PC_{Vi,Vi+t}^{NT}\;=L_{{}_{V_{i},V_{i+1}}}^{NT}\times \prod_{k=1}^{t-2}L_{V_{i+k}V_{i+(k+1)}}^{NT}\;\times L_{V_{i+t-1}V_{i+t}}^{NT}\ldots \ldots H2:non-real-time \end{array}\right.$$

where $L_{ViVj}^{RT}$ is the link connectivity for real-time traffic, $L_{ViVj}^{NT}$ is the link connectivity for non-real-time traffic, $PC_{Vi,Vi+t}^{RT}$ is the path connectivity for real-time traffic from node *V_i_* to any other node *V_i+t_* and $PC_{Vi,Vi+t}^{NT}$ is the path connectivity for non-real-time traffic from node *V_i_* to any other node *V_i+t_* in Equations (15) and (16). 

Foregoing in view, the constructed paths are node-disjoint paths which have no rendezvous point except source and destination. Node-disjoint paths are also preferred because they utilize most available network resources while avoiding the bottle necks by keeping energy balance. If an intermediate node fails in a node-disjoint path, only the path containing that failed node will be affected, thus maintaining the diversity of the routes intact with minimum impact. To avoid misuse of energy resources, we limit each sensor node to involve either in first-best hop decision or second-best hop decision, so that no sensor node is involved in constructing paths for both real-time and non-real-time traffic. Algorithm 3 provides details about the multi-disjoint path selection for real-time and non-real-time traffic.

After the construction of multi-disjoint paths, we need to divide our total paths for real-time and non-real-time traffic (i.e., out of N available paths, let us assume that there are *μ* paths that correspond to a probability of successfully delivering data to a destination) [46]. For real-time traffic, we need *τ* paths and for non-real-time traffic, we need *ϵ* paths, where the total traffic *μ = τ* + ε. Assuming that the traffic size of real-time and non-real-time data is known, we can easily calculate *τ* and ε. If *R_T_* represents the real-time traffic size and *N_T_* represents non-real-time traffic size, we can have:

(17) $$\tau =(\frac{R_{T}}{R_{T}+N_{T}})\mu$$

(18) $$\varepsilon =(\frac{N_{T}}{R_{T}+N_{T}})\mu$$

Moreover, the path connectivity from mobile sink $V_{SINK}$ to $V_{j}$ for real-time and non-real-time traffic can be expressed in Equations (19) and (20). The path information from mobile sink $V_{SINK}$ to $V_{j}$ for real-time and non-real-time traffic can be seen in Equations (21) and (22).

(19) $$PC_{V_{SINK}V_{j}}^{RT}\;=PC_{V_{SINK}V_{i}}^{RT}\times \prod_{k=0}^{t-2}L_{V_{i+k}V_{i+(k+1)}}^{RT}\;\times L_{V_{i+t-1}V_{i+t}}^{RT}$$

(20) $$PC_{V_{SINK}V_{j}}^{NT}\;=PC_{V_{SINK}V_{i}}^{NT}\times \prod_{k=0}^{t-2}L_{V_{i+k}V_{i+(k+1)}}^{NT}\;\times L_{V_{i+t-1}V_{i+t}}^{NT}$$

(21) $$PATH(V_{SINK},V_{j},*)=\{V_{SINK},V_{j},t+1\}=\{V_{SINK},V_{i}^{RT},\ldots V_{i+t-1}^{RT},V_{j}\}$$

(22) $$PATH(V_{SINK},V_{j},*)=\{V_{SINK},V_{j},t+1\}=\{V_{SINK},V_{i}^{NT},\ldots V_{i+t-1}^{NT},V_{j}\}$$

where *t* is the hops on the path between node $V_{i}$ to $V_{j}$ and *t* + 1 hops on the path between $V_{SINK}$ to $V_{j}$. 


**Algorithm 3: Optimal Multi-Disjoint Paths Selection for Real-Time and Non-Real-Time Traffic (Distinguishable Service)**

**Input:**
1.Formation of data set *D(S,N_s_,W/S,F_ST_,R_E_,B_S_,S/N)*
**Output:**
2.Optimal multi-disjoint paths for delay-sensitive and delay-tolerant traffic
**Begin:**
3.***for*** each awake node *V_i_ and V_j_* in network, ***do***4.  Determine *L_ViVj_* in terms of *TF, R_E_, B_S_, S/N* for first and second best hop decision criteria5.  ***if*** (delay sensitive traffic is needed), ***then*** //real-time data6.    $L_{ViVj}^{RT}=\underset{Vj\in N}{\max}\{\alpha TF_{ViVj}+\beta R_{E,Vj}+\gamma B_{S,Vj}+\sigma {\left(S/N\right)}_{ViVj}\}$7.  ***elseif*** (delay tolerant traffic is needed), ***then*** //non-real-time data8.    $L_{ViVj}^{NT}=\underset{Vj\in N}{2nd\max}\{\alpha TF_{ViVj}+\beta R_{E,Vj}+\gamma B_{S,Vj}+\sigma {\left(S/N\right)}_{ViVj}\}$9.  ***else*** Link connectivity cannot be established due to status transition (i.e., node sleeping)10.  ***end if***11.  Determine the path connectivity for both delay-sensitive and delay-tolerant traffic12.  ***if*** (delay-sensitive traffic is needed), ***then*** //real-time data13.    $PC_{V_{SINK}V_{j}}^{RT}\;=PC_{V_{SINK}V_{i}}^{RT}\times \prod_{k=0}^{t-2}L_{V_{i+k}V_{i+(k+1)}}^{RT}\;\times L_{V_{i+t-1}V_{i+t}}^{RT}$14.  ***elseif*** (delay tolerant traffic is needed), ***then*** //non-real-time data15.    $PC_{V_{SINK}V_{j}}^{NT}\;=PC_{V_{SINK}V_{i}}^{NT}\times \prod_{k=0}^{t-2}L_{V_{i+k}V_{i+(k+1)}}^{NT}\;\times L_{V_{i+t-1}V_{i+t}}^{NT}$16.  ***else*** link and path connectivity failed (i.e., path is no longer available)17.  ***end if***18.  Formulate real-time and non-real-time paths from total paths *μ*19.  ***if*** (delay sensitive traffic is needed), ***then*** //real-time data20.    total real-time paths will be $\tau *PATH(V_{SINK},V_{j},*)=\tau *\{V_{SINK},V_{i}^{RT},\ldots V_{i+t-1}^{RT},V_{j}\}$21.  ***else*** total non-real-time paths will be $\varepsilon *PATH(V_{SINK},V_{j},*)=\varepsilon *\{V_{SINK},V_{i}^{NT},\ldots V_{i+t-1}^{NT},V_{j}\};$22.  ***end if***23.  Select the optimal path using following expressions24.  $\max\{PC_{V_{SINK}V_{j}}^{RT},PC_{V_{SINK}V_{i}}^{RT}\times \prod_{k=0}^{t-2}L_{V_{i+k}V_{i+(k+1)}}^{RT\_updated}\;\times L_{V_{i+t-1}V_{i+t}}^{RT\_updated}\}$25.  $\max\{PC_{V_{SINK}V_{j}}^{NT},PC_{V_{SINK}V_{i}}^{NT}\times \prod_{k=0}^{t-2}L_{V_{i+k}V_{i+(k+1)}}^{NT\_updated}\;\times L_{V_{i+t-1}V_{i+t}}^{NT\_updated}\}$26.
***end for***



After the selection of optimal paths for real-time and non-real-time traffic, we need to define our routing strategy for data transmission and reception. The traditional opportunistic node connection routing only includes asynchronous working–sleeping cycle of sensor nodes which could lead to failed link connection sometimes, due to the sleeping mode of any forwarder node. Also, it does not support different type of data requirements in SGs, so in order to resolve this ambiguity, we need energy-efficient multi-disjoint path supporting opportunistic connection routing protocol. Algorithm 4 provide details about our routing strategy for delay sensitive and delay tolerant traffic in EMOR.


**Algorithm 4: Routing Strategy for Delay Sensitive and Delay Tolerant Traffic in EMOR**

**Input:**
1.OCRG, spanning tree formation, optimal path connectivity for distinguishable service
**Output:**
2.Energy efficient routing protocol based on optimal path connectivity for distinguishable service
**Begin:**
3.Assuming that NAN sensing data from node *V_m_* is forwarded to *V_SINK_* using optimal path4.When an intermediate node *V_r_* receives the sensing data in NAN:5. ***if*** (the successor node *V_s_* of node *V_r_* is in working mode), ***then***6.   Forward the sensing data to *V_s_*7.    ***if*** (*V_s_* knows optimal path towards *V_SINK_* in its routing table), ***then***8.      *V_s_* receives the sensing data from *V_r_* and forwards the data on optimal path;9.   ***elseif*** (*V_s_* does not know optimal path towards *V_SINK_* in its routing table), ***then***10.      ***if*** (*V_s_* has neighbors on the spanning tree), ***then***11.        ***for*** (each working neighbor *V_i_* of *V_s_* node), ***do***12.          ***if*** (delay sensitive traffic is needed), ***then***13.            Calculate $L_{VsVi}^{RT}$ for real-time data14.            Determine $PC_{VsVi}^{RT}$ for real-time data based on $L_{VsVi}^{RT}$15.          ***elseif*** (delay tolerant traffic is needed), ***then***16.            Calculate $L_{VsVi}^{NT}$ for non-real-time data17.            Determine $PC_{VsVi}^{NT}$ for non-real-time data based on $L_{VsVi}^{NT}$18.          ***end if***19.        ***end for***20.        Select the optimal path using Algorithm 2 and 3 21.      ***elseif*** (*V_s_* has no neighbors on the spanning tree), ***then***22.        Stop forwarding the data23.      ***end if***24.   ***end if***25.***elseif*** (the successor node *V_s_* of node *V_r_* is sleeping), ***then***26.   Search for other neighbors or wait until *V_r_* wakes up27.
***end if***



**Figure 6:**
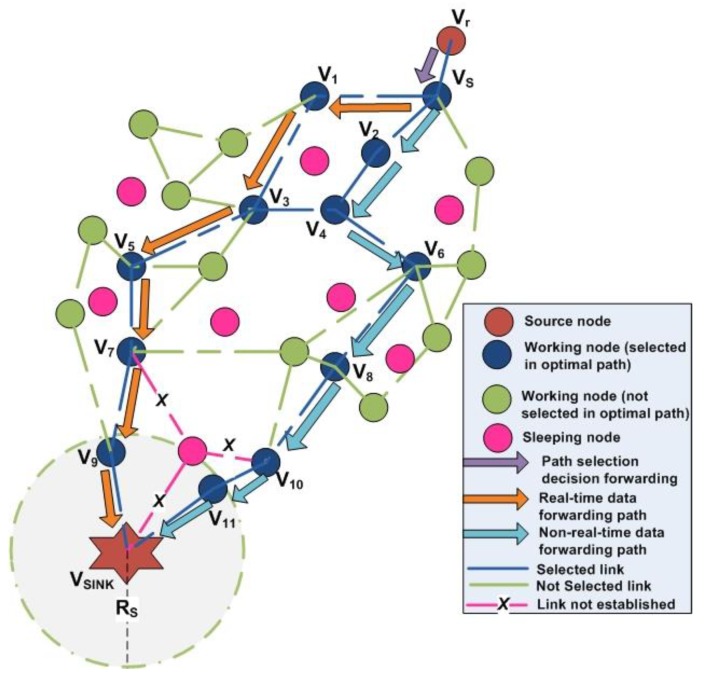
Multi-disjoint path selection and routing strategy for real-time and non-real-time traffic.

Based on the routing strategy algorithm for delay-sensitive and delay-tolerant traffic in EMOR, the detail process of a source node forwarding its sensed data to the mobile sink can be depicted from Figure 6. When a random node *V_s_* receives the data from one of its neighboring nodes, it follows the optimal multi-disjoint path selection for delay sensitive and delay tolerant traffic. Node *V_1_* receives the delay sensitive traffic as it has the maximum link connectivity with *V_s_* and node *V_2_* receives the delay tolerant traffic as it has the second maximum link connectivity value with V_s_. During the transmission of delay-sensitive and delay-tolerant traffic in the network, we have to ensure that the nodes can be part of the optimal path towards mobile sink if they offer higher link connectivity. Although the nodes appearing as green in Figure 6 are working, they do not offer max or second max link connectivity, so they are not part of the delay-sensitive or delay-tolerant traffic paths. Moreover, the nodes appearing as pink in Figure 6 are sleeping nodes, so they could not be involved in the processes of OCRG, spanning tree, and optimal paths towards mobile sink. 

After the path connectivity for delay-sensitive and delay-tolerant traffic is established, we have to update the path connectivity value by comparing the path connectivity value in previous rounds with the path connectivity value in the current round of communication and select the maximum of the two values. In this way, we will be able to acquire the optimal path from any working sensor node to the mobile sink.

## 5. Performance Evaluation of EMOR

In this section, we describe the simulation environment and simulation results with reference to performance metrics used for EMOR. 

### 5.1. Simulation Environment

In order to validate the effectiveness of EMOR in NAN, we evaluate the performance of EMOR in MATLAB 2018 simulator [56] and the parameters used in this simulation are defined in Table 3 as:

We conducted a set of simulation experiments for EMOR protocol and performed a comparative analysis of EMOR with several other protocols used in sensor networks. Our simulation environment includes a network field of 500 m × 500 m in which 200 sensors were deployed randomly. We have assumed that all sensor nodes are independent and identical with a communication range of 20 m. We have fixed the duration of the data collection period in our experiments to check the impact of several other parameters on EMOR’s performance and to compare that performance with various algorithms used in sensor networks. The classical data transmission algorithms like Extremely Opportunistic Routing (ExOR) [19] improves the reliability of data forwarding by exploiting the broadcasting nature of wireless medium in such a way that allows multiple neighboring nodes to overhear the ongoing transmission and participate in data forwarding, whereas probabilistic and opportunistic flooding algorithm (POFA) [57] achieves the target reliability on each hop of data forwarding by adopting the controlled transmissions but at the cost of higher energy consumption and increased number of hopes. Venkatesha et al. in [58] presented reliable proliferation routing with low duty cycle (RPRDC) by integration of concepts like randomized dispersity, forwarding, and reliable path finder to improve the packet delivery ratio. Yang et al. in [22] presented a data collection model based on opportunistic node connection (DCBONC) in wireless sensor networks. DCBONC calculates the optimal path from the mobile sink to every sensor node in the graph by formulation of a spanning tree. DCBONC also utilizes a routing protocol which adapts to different sensor nodes statutes to improve the reliability of data transmission. DCBONC depicts improvement in performance in parameters like packet delivery ratio (PDR), energy consumption, and network lifetime. Foregoing in view, most of the classical algorithms used in sensor networks, consider unreliable wireless links due to lack of link quality parameter. Most of the algorithms do not include multipath links for data forwarding except ExOR and POFA. In addition to it, no sensor network algorithm developed so far has contributed towards SGs NAN while focusing on real-time and non-real-time traffic requirements and efficiently managing the energy resources.

Moreover, the traditional data forwarding algorithms and schemes do not incorporate OCRG in calculating the optimal path towards the sensor nodes, so an algorithm is needed which utilizes the concept of OCRG, highlights the link quality aspect, and also demonstrates adherence to accomplish the real-time and non-real-time requirements of SGs NAN while efficiently managing the energy resources. Therefore, we propose an energy efficient multipath opportunistic routing protocol for smart grids NAN. The performance comparison of EMOR with that of ExOR, POFA, RPRDC, and DCBONC is completed with reference to performance metrics like link connectivity, PDR, energy consumption, network lifetime, and average end-end delay.

### 5.2. Results

#### 5.2.1. Link Connectivity

**Figure 7:**
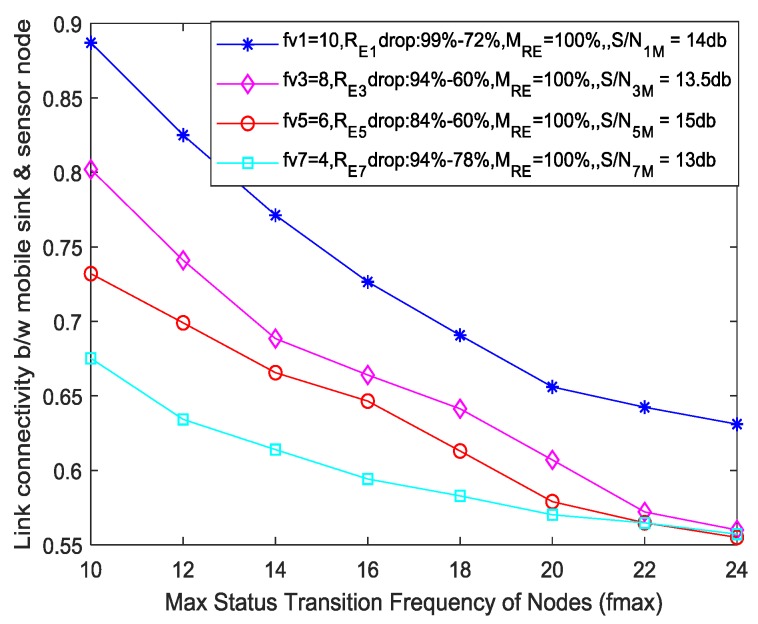
Link connectivity between different sensors and mobile sink (based on time frequency (TF) parameter, residual energy, buffer size, and link quality factor) against maximum status transition frequency of nodes in a spanning tree.

As from the Section 4.3, “Construction of Opportunistic Connection Random Graph”, we know that link connectivity between a mobile sink node and any sensor node or between sensor nodes depend on several parameters like time-frequency parameter, residual energies of both nodes, remaining buffer size of both nodes, and link quality factor. Two scenarios are designed to conduct simulations to evaluate link connectivity. In the first scenario, the link connectivity is calculated between mobile sink node and sensor node. In this scenario, we assume that mobile sink node is always in working status but the sensor node, depending on its working–sleeping cycle, performs certain status transitions, so the link connectivity if calculated between the mobile sink node and any sensor node, depends on the time frequency (TF) parameter of that sensor node, residual energy of that sensor node, remaining buffer size of that sensor node, and signal-to-noise ratio between that sensor node and mobile sink node. In this scenario, we consider multiple sensor nodes with different values of TF, residual energy, and link quality factor to evaluate their link connectivity against mobile sink node. Also, as we have assumed that the mobile sink node always remains in the working state, so its energy is always 100% and the total working time of sensor nodes is 75% of data collection duration *T_CP_*. Figure 7 depicts that the overall link connectivity decreases with the increase in max status transition frequency. The main reason will be the reduction in residual energies of nodes and the decreased status transition frequencies of individual nodes. For nodes with higher status transition frequency, the link connectivity is higher. Foregoing in view, the higher status transition of a node will create increased opportunities of sensing data sharing between that node and mobile sink node, thus improving the link connectivity between them but at the same time, for higher status transitions of a node, there will be a corresponding decrement in the residual energy as the power is being consumed every time (waking up and sleeping) a node undergoes a status transition as shown in Figure 2.

In the second scenario, the link connectivity is calculated between different adjacent sensor node pairs. In this scenario, we assume that all the sensor nodes, depending on their working–sleeping cycle, perform certain status transitions, so the link connectivity if calculated between adjacent sensor node pairs, depending on the TF parameter of those sensor nodes, the residual energy of those sensor nodes, the remaining buffer size of those sensor nodes, and signal-to-noise ratio between that node pair. In this scenario, we consider multiple sensor nodes with different values of TF, residual energies, and link quality factors to evaluate their link connectivity between them.

**Figure 8:**
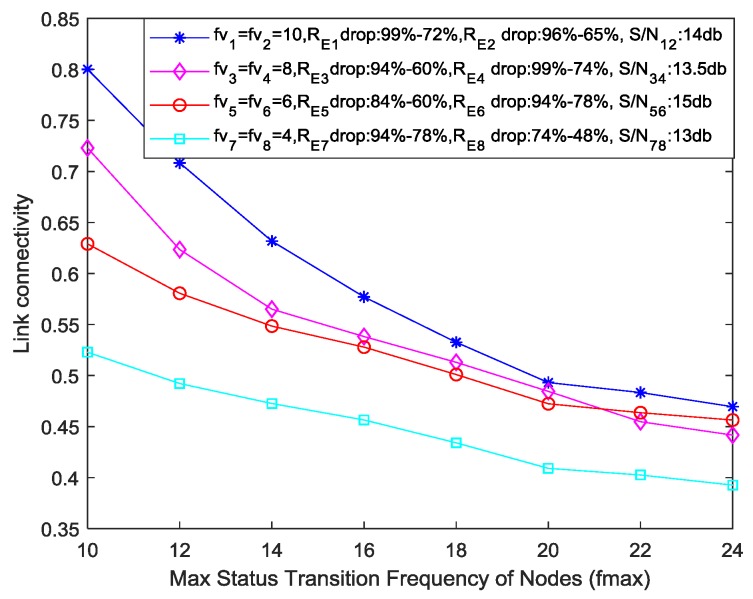
Link connectivity between different adjacent sensors (based on the TF parameter, residual energy, buffer size, and link quality factor) against maximum status transition frequency of nodes in the spanning tree.

Figure 8 depicts that the overall link connectivity decreases with the increase in maximum status transition frequency. The main reason will be the decreasing status transition frequencies of different node pairs and cumulative reduction in residual energies used for simulation. We can see from Table 4 that when node pair status transition frequency decreases from 10 to 4, there is a corresponding effect on the overall link connectivity between each node pair. The difference between the first scenario and second scenario is the mobile sink. When we use a mobile sink and a sensor node for data sharing, we have to consider only the effect of sensor node parameters on the link connectivity whereas in the case of adjacent node pairs, we have to consider the effect of parameters related to the node pair. Keeping in view the weights used in Table 3, we can analyze the decrease in overall connectivity in Figure 8, as compared to link connectivity calculated in the first scenario (i.e., for fv1 = fv2 = 10, we can see a reduction of 25% in the impact of TF parameter for link connectivity in the second scenario, for fv3 = fv4 = 8, we have 40% decrease in the impact of TF parameter for link connectivity). In case of fv5 = fv6 and fv7 = fv8, the effect of TF-parameter in the link connectivity calculation is reduced by 55% and 70%, respectively. Using Equations (4) and (6) and the assumption that total working time of sensor nodes is 75% of the data collection duration, the effect of the TF parameter on link connectivity calculation can be determined by the following expression,

(23) $$TF_{ViVj}\;=\left(\frac{F_{STVi}}{F_{STmax}}\times \frac{\left(0.75*T_{CP}\right)}{T_{CP}}\right)\left(\frac{F_{STVj}}{F_{STmax}}\times \frac{\left(0.75*T_{CP}\right)}{T_{CP}}\right)$$

(24) $$TF_{ViVSINK}=\left(\frac{F_{STVi}}{F_{STmax}}\times \frac{\left(0.75*T_{CP}\right)}{T_{CP}}\right)$$

(25) $$\frac{TF_{ViVj}}{TF_{ViVSINK}}\;=\left(\frac{F_{STVi}}{F_{STmax}}\times 0.75\right)$$

where the ratio of the TF parameter for adjacent node pairs to the TF parameter between mobile sink and single sensor node in Equation (25), is used to study the impact or influence of the TF parameter on the link connectivity calculation in the second scenario. In addition to it, the effect of residual energy on the link connectivity calculation can be determined using Equations (26) and (27):

(26) $$R_{E(1^{st}scenario)}=\beta R_{Ei};R_{E(2^{nd}scenario)}=\beta (R_{Ei}*R_{Ej})$$

(27) $$\frac{R_{E(2^{nd}scenario)}}{R_{E(1^{st}scenario)}}=R_{Ej}$$

The residual energy ratio in Equation (27) can be used to study the impact of residual energy on the link connectivity calculation in the second scenario. It is quite obvious that the only difference in residual energy impact on link connectivity is residual energy of node *j* in the node pair.

For example, if the residual energy of a node is 0.94 in the first scenario and the residual energies of the adjacent node pair are 0.94 and 0.74, subject to the condition that the node used in the first scenario belongs to the node pair in the second scenario, then the percentage change in the impact of R_E_ on overall link connectivity will be 26% decreased according to Equation (27) and Table 5. It should also be noted that during our simulation, the effect of buffer size for all nodes is not considered as the percentage change in overall link connectivity because the buffer size was negligible.

#### 5.2.2. Packet Delivery Ratio (PDR)

**Figure 9:**
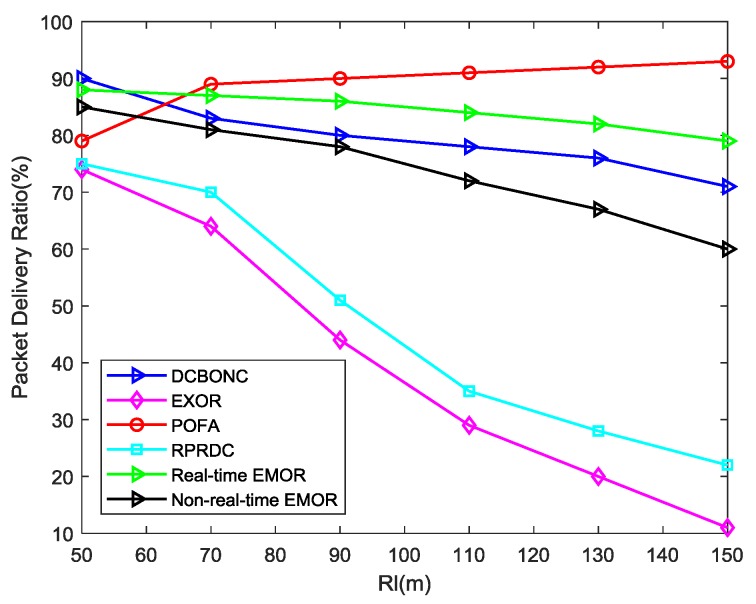
Packet delivery ratio against long radio range of mobile sink where number of nodes (*N*) = 1000. DCBONC: Data Collection Algorithm based on Opportunistic Node Connection; EXOR: Extremely Opportunistic Routing; POFA: Probabilistic and Opportunistic Flooding Algorithm; RPRDC: Reliable Proliferation Routing with Low Duty Cycle; EMOR: Energy Efficient Multi-Disjoint Path Opportunistic Node Connection Routing Protocol.

The metric PDR can be defined as the ratio of the packets received by the mobile sink node to the packet sent by the sensor nodes. We have designed two scenarios to perform simulation for the evaluation of PDR. In the first scenario, PDR is evaluated against long radio range *R_l_* of the mobile sink node whereas in the second scenario, the performance of the PDR was measured against network density. In order to set up the simulation environment for PDR, we need to set a benchmark for the packet successfully transmitted and packet dropped due to certain reasons. If the node finds one of its neighboring nodes is in working mode or if the data packet is forwarded between adjacent sensor nodes for which both of the sensor nodes are in working mode, we can consider it as the benchmark of a packet successfully transmitted; whereas if the node cannot find the forwarder due to asynchronous working–sleeping cycle strategy or if the data packet is forwarded between adjacent sensor nodes and one of the node experiences status transition (i.e., working to sleeping mode, thus causing transmission failure), then we will consider it as the benchmark for a packet drop. Figure 9 shows that PDR for POFA [57] increases with the increase in long radio range *R_l_* but for schemes like DCBONC [22], RPRDC [58], ExOR [19] and EMOR, the PDR decreases with the increase in long radio range *R_l_*. The reason for the increase in PDR for POFA can be explained in terms of unchanged network density. When *R_l_* increases, more nodes will be part of the data collection process but as the total number of nodes remains the same, it results in the increase of hops for all the sensor nodes in the data collection scope. This increase in number of hops will eventually decrease the success rate of transmission and delivering the sensory data to mobile sink node correspondingly. However, the sensory data will be forwarded to mobile sink node through multiple paths in POFA, thus increasing the possible paths towards mobile sink node and improving the success rate of data transmission and reception.

It is evident from Figure 9 that our proposed scheme EMOR incorporates real-time and non-real-time disjoint paths and the performance of both is better than POFA (for long radio range *R_l_* up to 71%). For real-time EMOR, the performance is better than ExOR, RPRDC, and DCBONC throughout the graph as *R_l_* expands due to the reason that the proposed scheme always forwards the sensing data along its optimal path which is based on maximum value of path connectivity. In order to strengthen our basis for improvement in PDR, the link connectivity (in EMOR) depends on several factors like time-frequency parameter, residual energies of sensor nodes, buffer capacity of sensor nodes, and link quality factor between sensor nodes and mobile sink node. Furthermore, the proposed scheme also utilizes the routing protocol which is based on the concept of multi-disjoint path selection for real-time and non-real-time data, in which real-time EMOR follows the criteria of the first best hop and non-real-time EMOR follows the criteria of the second best hop, thus improving the PDR.

**Figure 10:**
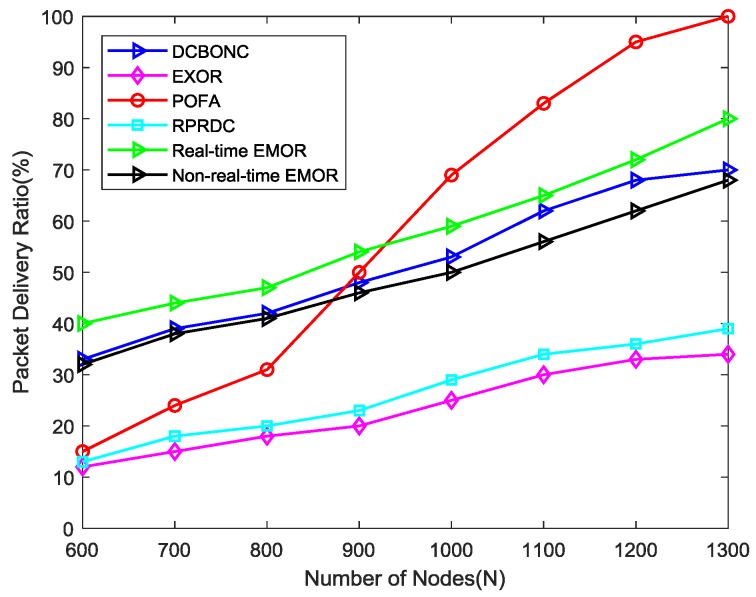
Packet delivery ratio (PDR) against network density with long radio range of mobile sink *R_l_* as 100 m.

The PDR against network density graph can be seen in Figure 10, in which PDR increases with an increase in the number of nodes in the network for all of the schemes used in the simulation. The number of nodes in the working mode will increase due to increased network density, therefore we can see an increase in the overall PDR. The proposed scheme achieves 138%, 110%, 10.5%, and 36.39% improvement in PDR over ExOR, RPRDC, DCBONC, and POFA (up to 925 nodes), respectively. The improvement in PDR for POFA beyond 900 nodes is better than our proposed scheme due to the fact that there are multiple forwarders in POFA to achieve the target reliability value. When the number of connections increases, the possibility of achieving that target reliability value also increases, thus improving the PDR. In RPRDC, the routing is segmented into random dispersion and reliability path exploration with the consideration of variables such as residual energy, packet reception rate, and link quality but it does not provide enough information between sink node and sensor nodes for achieving better PDR. ExOR [19] works on the metric expected transmission times (ETX) in such a way that the node with a minimum value of ETX will be selected for forwarding data towards the sink node and does not include information related to path connectivity, thus leading to decreased PDR.

#### 5.2.3. Energy Consumption

**Figure 11:**
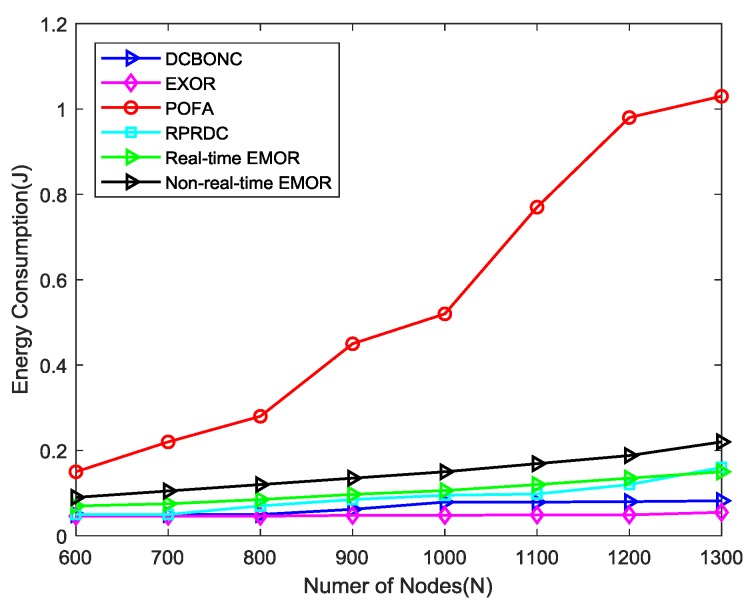
Energy consumption against network density, with long radio range of mobile sink *R_l_* as 100 m.

In order to evaluate the performance of energy consumption, we have designed two scenarios using our simulation parameters. In the first scenario, we consider that the source (sensor) node is forwarding its sensing data to the mobile sink, so we evaluate the energy consumption after contemplating this consideration. In the second scenario, we evaluate the total energy consumption against the simulation time. Figure 11 depicts that energy consumed during the sensing data forwarding by a sensor node to mobile sink changes by network density. We have assumed that the maximum distance between the sensor node and the mobile sink can be *3R_l_/4* just for the sake of this experiment.

We can observe from the Figure 11 that energy consumption increases when the number of nodes increases in the network or when network density increases. The energy consumption by the POFA scheme is more obvious than in any other scheme including our proposed scheme. The energy consumption in POFA can be explained in a way that when network density increases, the number of hops used for data delivery between the sensor node and mobile sink increases too, so the compilation of energy consumed by every hop results in the overall increase of energy consumption. Furthermore, the energy consumed by the proposed scheme is slightly higher than DCBONC and ExOR schemes. The reason is when a node sleeps in the neighborhood of a source node due to asynchronous working–sleeping cycle strategy, which was used as a data forwarding node towards mobile sink in the past, several processes like optimal path calculation, optimal path selection for real-time/non-real-time data and multipath opportunistic routing protocol are repeated for neighbors which are awake, thus resulting in a slight increase in energy consumption but still far better than POFA scheme. The energy consumption for non-real-time EMOR is slightly higher than real-time EMOR as we select the second best hop for our path connectivity in non-real-time EMOR in which it becomes more probable that the PDR for the second best hop path will be slightly less, and energy consumption will be slightly more than that of real-time EMOR.

**Figure 12:**
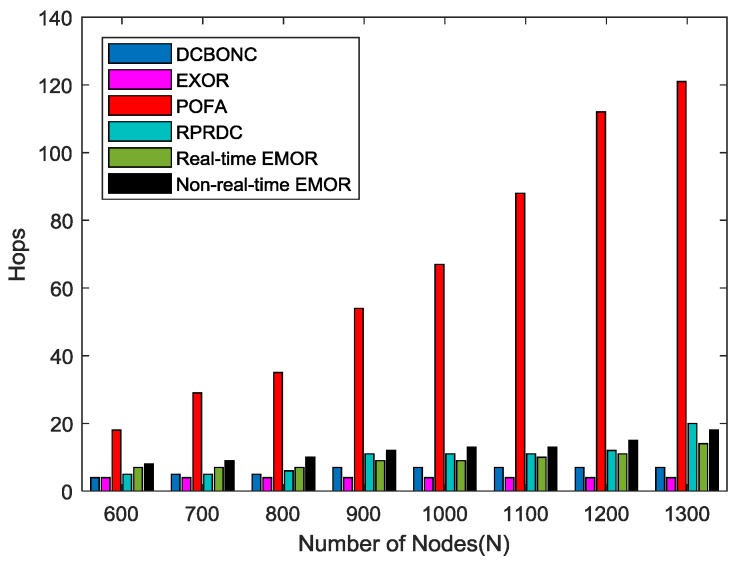
The variation in number of hops against network density, with long radio range of mobile sink *R_l_* as 100 m.

Figure 12 shows the increase in number of hops when the network density is increased. It is evident that the increase in number of hops in the POFA scheme are higher than in any other scheme. The number of hops for non-real-time EMOR are more than that of real-time EMOR because of the fact that non-real-time path selection is based on the second best hop decision in which the second-to-highest priority is given to the non-real-time path. The probability of a node having a certain status transition is higher in the non-real-time path than in the real-time path (depending on the TF parameter). Moreover, the immediate nodes present near the mobile sink are already loaded as the traffic convergence nodes, so in order to avoid them, we have to select alternative paths (resulting in increased hops), which means that as the network density increases, the number of hops for non-real-time path selection are increased more than the real-time path.

**Figure 13:**
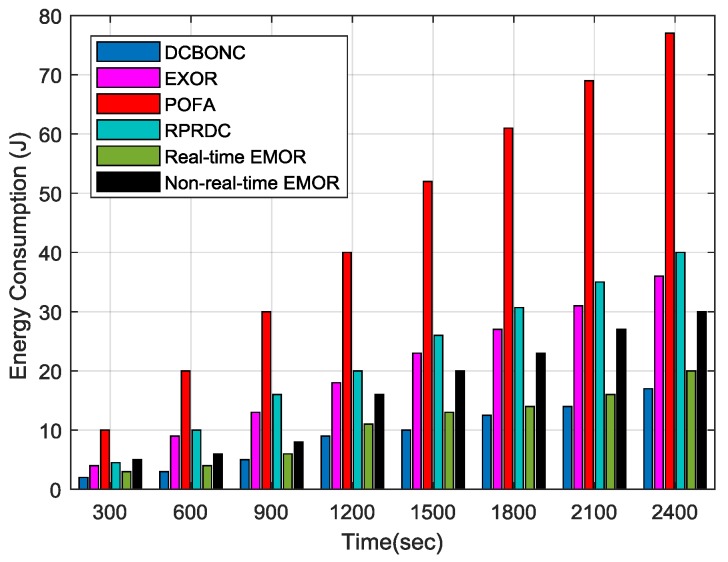
Total energy consumption against network simulation time, with *Rl* as 100 m and *N* = 1000.

In the second scenario, we conducted the total energy consumption against the network density simulation. It is quite obvious from Figure 13 that total energy consumed increases as the simulation time increases. Here, we have considered only the total energy consumed during the data collection phase by the mobile sink and energy consumed during the sensing data delivery by source node, regardless of the condition that the sensing data is successfully delivered to the mobile sink or not. In comparison to proposed scheme, ExOR consumes 84% more energy, POFA consumes 307% more energy and RPRDC consumes 107% more energy, whereas in the case of DCBONC, the energy consumption of the proposed scheme EMOR is slightly higher (i.e., 18% more). The main reason is that in DCBONC, the probe message forwarding mechanism in the data collection period consumes less energy due to the incorporation of EOH [22]. Furthermore, the spanning tree is constructed and broadcasted by mobile sink so that the source node could obtain the max path connectivity information beforehand, thus saving a lot of energy consumed by selecting the forwarders. However, the additional energy consumed during the selection of optimal paths for real-time and non-real-time traffic is the difference in total energy of EMOR and DCBONC.

#### 5.2.4. Average End-End Delay

**Figure 14:**
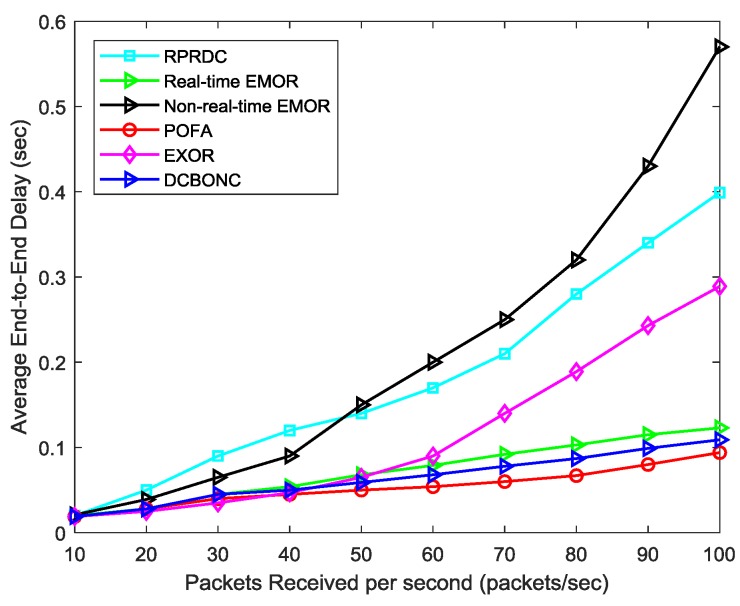
Average end-end delay against packets received.

Average end-end delay is an important metric in evaluating the performance of energy efficient QoS-based routing protocols. Here, we have conducted the simulation in terms of average packet delay. Figure 14 depicts the average packet delay against packet arrival rate for several different schemes. In this experiment, we can observe the change in delay with respect to change in packet arrival rate. We measured the average packet delay for both real-time and non-real-time traffic in our proposed scheme to validate whether EMOR can successfully differentiate between them or not. From the graph, it is quite clear that high priority is given to real-time traffic due to the reason that real-time traffic is given absolute preferential treatment over low priority non-real-time traffic in EMOR design, thus resulting in low end-end delay. Real-time EMOR performs much better than non-real-time EMOR in comparison to other schemes because of the reason that we use the queueing model for SGs NAN environment. The average packet delay in POFA is much less as it incorporates the multipath forwarding strategy depending on its target reliability. Additionally, DCBONC performance is slightly better than real-time EMOR due to several reasons (i.e., decision variables for calculating the link connectivity and path connectivity in DCBONC are very few). Furthermore, there is no differentiation between the traffic types in DCBONC, thus resulting in lower average packet delay. Non-real-time EMOR performs better than RPRDC up to 48 packets received per second and then achieves a higher delay due to the queueing model being employed in our design.

#### 5.2.5. Network Lifetime

**Figure 15:**
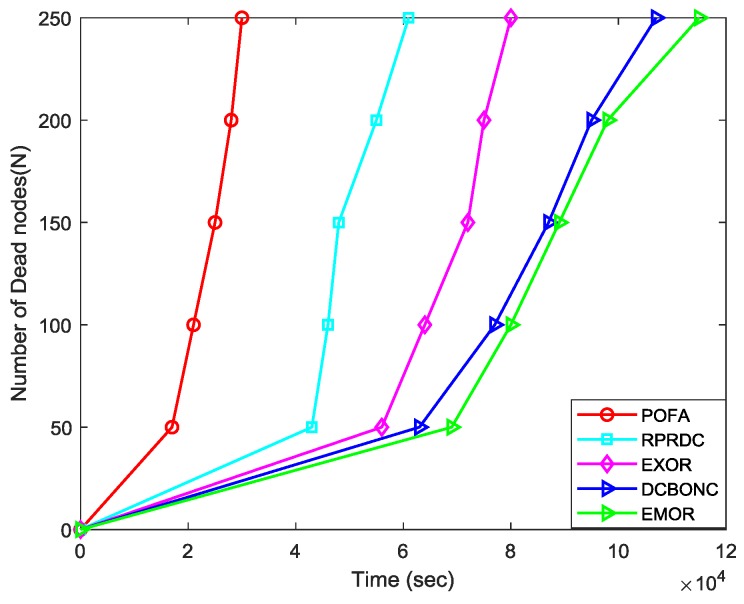
Network life time (number of dead nodes against simulation time) with *R_l_* = 90 m and *N* = 850.

Here we conducted the simulation with the condition that our mobile sink can launch multiple data collection tasks anywhere within the network. The performance of network lifetime can be evaluated in terms of number of dead nodes for which according to [59] Tunca et al. have highlighted that network lifetime for WSNs can be defined in terms of the time when 25% of the nodes present in the network have no residual energy left to continue their data delivery tasks. Figure 15 demonstrates the network lifetime performance of different schemes against their simulation time. It can be seen that the number of dead nodes increases as the simulation time increases. In this simulation, we have assumed that the data collection period for mobile sink is 600 seconds and our mobile sink can start the data collection task randomly within the network.

In POFA, the node dies faster than any other scheme due to the reason that POFA focuses more on the reliability of data transmission at the expense of energy consumption, so it reaches the 25% dead node mark faster than any other scheme. It is evident from the graph that the performance of EMOR is better than all schemes including DCBONC. The main reason is the calculation of optimal paths for nodes in advance and employment of flexible routing protocol. The slight difference between DCBONC and real-time EMOR can be explained by the fact that decision variables for calculation of optimal paths in EMOR are more effective than that of DCBONC. In DCBONC, only TF-parameter is used in calculation of optimal paths whereas in EMOR, we use the TF parameter, residual energy, buffer size, and link quality factor to achieve better network lifetime. Moreover, the energy consumed per node is less in EMOR in comparison to other schemes due to the fact the EMOR can change the data forwarding paths adaptively, especially in the low connectivity areas of the network.

## 6. Discussion

We have introduced the energy efficient multipath opportunistic routing protocol for SGs NAN environment. The previous opportunistic routing schemes were designed without consideration of service differentiation and multiple decision variables for calculation of optimal path connectivity. In EMOR, we utilized the concepts of multiple decision variables in optimal path connectivity and service differentiation in optimal path selection for real-time and non-real-time traffic. The proposed design of EMOR could be applicable to the following different approaches in future:

(1) Incorporation of relay nodes and finding the optimum number of relay nodes to support network lifetime.
(2) Design an energy efficient multipath opportunistic routing protocol for wireless cognitive sensor networks.
(3) Keeping in view the variable design of sensors and network architectures, heterogeneous clustering could be used with EMOR for wide-range connectivity.
(4) How to increase the network lifetime of WSNs having sensor nodes which follow asynchronous working–sleeping cycle strategy by achieving a tradeoff between EMOR and energy balancing routing protocols.
(5) How to reduce the network end-end delay of WSNs having sensor nodes which follow asynchronous working–sleeping cycle strategy by achieving a tradeoff between POFA and real-time EMOR.
(6) Unmanned Aerial Vehicle (UAV) sensor networks, Internet-of-Things (IOT) enabled home energy systems, cyber-physical systems, etc.
(7) Energy harvesting wireless sensor networks [60].

## 7. Conclusions

In this paper, we proposed an energy-efficient multipath opportunistic routing protocol for wireless sensor networks, which can be used in neighborhood area network of smart grids. In this proposed scheme, the mobile sink launches the data collection task anywhere and anytime in the network by broadcasting its SID in the tag message. Upon receiving the tag message, the sensor nodes start formulating their working–sleeping schedule and then forward a probe message to the mobile sink as a response to the tag message. The probe message contains the source information, working–sleeping schedule, and status transition frequency of that sensor node. When the mobile sink receives this probe message, it constructs an opportunistic connection random graph and calculates the optimal path from itself to each sensor node. The optimal path formation is based on the dataset of link connectivity and path connectivity, which includes time-frequency parameter, residual energy, buffer capacity, and link quality factor. After calculation of the optimal path, the first spanning tree is generated in which mobile sink is considered as the root node. Keeping in view the nature of NAN traffic in smart grids, we performed multi-path selection for real-time and non-real-time traffic based on the first and second best possible decisions for link connectivity and path connectivity. The second spanning tree after the selection of real-time and non-real-time path was generated and optimal paths for real-time and non-real-time traffic were calculated. Consequently, we designed the routing protocol based on the optimal paths for real-time and non-real-time traffic of sensor nodes in the spanning tree while focusing on different working–sleeping cycle strategies of sensor nodes.

The possible future work could be incorporation of networks like cloud computing and fog computing with EMOR in which the high computation needs of the mobile sink in the sensor network can be fulfilled using fog computing nodes bridged with cloud. Additionally, the novel concept could be applied to cognitive radio sensor networks to deal with problems like predictive channel assignments and opportunistic spectrum access. Moreover, supervised machine learning techniques can also be used with our proposed scheme to investigate the performance improvements in OCRG and spanning tree formation, predictive changes in the real-time and non-real-time traffic trends (including volumes, faults, power surges, etc.).

## Figures and Tables

**Table 1:** Format of probe message in data collection scope.

Fields	Source ID	Working–Sleeping Schedule	Status Transition Frequency	Neighbor IDs	Sink ID	Forwarders ID	Expected Optimal Hops (EOH)
Length (bits)	10	15	10	10	10	60	5

**Table 2:** Path connectivity from V_SINK_ to each sensor node in the spanning tree.

Step	[v1]	[v2]	[v3]	[v4]	[v5]	[v6]	[v7]	[v8]	[v9]	[v10]
Initialization	PC_V1_	PC_V2_	PC_V3_	0	0	0	0	0	0	0
1	PC_V1_	PC_V2_	PC_V3_	PC_V1 *_L_V1V4_	PC_V3 *_L_V3V5_	0	0	0	0	0
2	Max{ PC_V1_, PC_V2 *_ L_V2V1_}	Max{ PC_V2_, PC_V1 *_ L_V1V2,_ PC_V3 *_ L_V3V2_}	Max{ PC_V3_, PC_V2 *_ L_V2V3_}	PC_V1 *_ L_V1V4_	PC_V3 *_L_V3V5_	PC_V4 *_ L_V4V6_	PC_V2 *_ L_V2V7_	PC_V5 *_ L_V5V8_	0	0
3	Max{ PC_V1_, PC_V2 *_ L_V2V1_}	Max{ PC_V2_, PC_V1 *_ L_V1V2,_ PC_V3 *_ L_V3V2_}	Max{ PC_V3_, PC_V2 *_ L_V2V3_}	Max{ PC_V1 *_ L_V1V4_, PC_V2 *_ L_V2V4_}	Max{ PC_V3 *_ L_V3V5_, PC_V2 *_ L_V2V5_}	PC_V4 *_ L_V4V6_	PC_V2 *_ L_V2V7_	PC_V5 *_ L_V5V8_	PC_V7 *_ L_V7V9_	PC_V8 *_ L_V8V10_
4	Max{ PC_V1_, PC_V2 *_ L_V2V1_}	Max{ PC_V2_, PC_V1 *_ L_V1V2,_ PC_V3 *_ L_V3V2_}	Max{ PC_V3_, PC_V2 *_ L_V2V3_}	Max{ PC_V1 *_ L_V1V4_, PC_V2 *_ L_V2V4_}	Max{ PC_V3 *_ L_V3V5_, PC_V2 *_ L_V2V5_}	Max{ PC_V4 *_ L_V4V6_, PC_V2 *_ L_V2V6_}	PC_V2 *_ L_V2V7_	Max{ PC_V5 *_ L_V5V8_, PC_V2 *_ L_V2V8_}	PC_V7 *_ L_V7V9_	PC_V8 *_ L_V8V10_
5	Max{ PC_V1_, PC_V2 *_ L_V2V1_}	Max{ PC_V2_, PC_V1 *_ L_V1V2,_ PC_V3 *_ L_V3V2_}	Max{ PC_V3_, PC_V2 *_ L_V2V3_, PC_V1 *_ L_V1V3_}	Max{ PC_V1 *_ L_V1V4_, PC_V2 *_ L_V2V4_}	Max{ PC_V3 *_ L_V3V5_, PC_V2 *_ L_V2V5_}	Max{ PC_V4 *_ L_V4V6_, PC_V2 *_ L_V2V6_, PC_V1 *_ L_V1V6_}	PC_V2 *_ L_V2V7_	Max{ PC_V5 *_ L_V5V8_, PC_V2 *_ L_V2V8_}	Max{ PC_V7 *_ L_V7V9_, PC_V6 *_ L_V6V9_}	PC_V8 *_ L_V8V10_
6	Max{ PC_V1_, PC_V2 *_ L_V2V1_}	Max{ PC_V2_, PC_V1 *_ L_V1V2,_ PC_V3 *_ L_V3V2_}	Max{ PC_V3_, PC_V2 *_ L_V2V3_, PC_V1 *_ L_V1V3_}	Max{ PC_V1 *_ L_V1V4_, PC_V2 *_ L_V2V4_}	Max{ PC_V3 *_ L_V3V5_, PC_V2 *_ L_V2V5_, PC_V7 *_ L_V7V5_}	Max{ PC_V4 *_ L_V4V6_, PC_V2 *_ L_V2V6_, PC_V1 *_ L_V1V6_, PC_V7 *_ L_V7V6_}	Max{ PC_V2 *_ L_V2V7_, PC_V5 *_ L_V5V7_, PC_V6 *_ L_V6V7_}	Max{ PC_V5 *_ L_V5V8_, PC_V2 *_ L_V2V8_}	Max{ PC_V7 *_ L_V7V9_, PC_V6 *_ L_V6V9_}	Max{ PC_V8 *_ L_V8V10_, PC_V9 *_ L_V9V10_}
7	Max{ PC_V1_, PC_V2 *_ L_V2V1_}	Max{ PC_V2_, PC_V1 *_ L_V1V2,_ PC_V3 *_ L_V3V2_}	Max{ PC_V3_, PC_V2 *_ L_V2V3_, PC_V1 *_ L_V1V3_}	Max{ PC_V1 *_ L_V1V4_, PC_V2 *_ L_V2V4_}	Max{ PC_V3 *_ L_V3V5_, PC_V2 *_ L_V2V5_, PC_V7 *_ L_V7V5_}	Max{ PC_V4 *_ L_V4V6_, PC_V2 *_ L_V2V6_, PC_V1 *_ L_V1V6_}	Max{ PC_V2 *_ L_V2V7_, PC_V5 *_ L_V5V7_, PC_V6 *_ L_V6V7_}	Max{ PC_V5 *_ L_V5V8_, PC_V2 *_ L_V2V8_, PC_V7 *_ L_V7V8_}	Max{ PC_V7 *_ L_V7V9_, PC_V6 *_ L_V6V9_, PC_V10*_L_V10V9_}	Max{ PC_V8 *_ L_V8V10_, PC_V9 *_ L_V9V10_, PC_V7 *_ L_V7V10_}

**Table 3:** Simulation parameters.

Parameters	Values
Network Size	500 × 500 m^2^
Number of the Mobile Sink	1
Number of Sensors	200
Mobility Pattern	Randomly
Duration for a Data Collection Period	600 s
Communication Range for Sensor Nodes	20 m
(Data + Overhead) Packet Size	1024 bits
Probe Message Size	120 bits
Transmit Power	15 mW
Receive Power	13 mW
Medium Access Control (MAC) Layer	IEEE 802.11
Max Buffer size	512 K-bytes
Target Reliability in Probabilistic and Opportunistic Flooding Algorithm (POFA)	0.6
Initial Energy of Nodes	2.5 J
Buffer Threshold	1024 bits
$E_{elec}$	20 × 10^−7^ J/bit
$\varepsilon_{amp}$	10 × 10^−9^ J/bit/m^2^
Weights (α, β, γ and σ) respectively	0.4, 0.3, 0.1, 0.2

**Table 4:** Effect of TF parameter on link connectivity.

Parameter	Status Transition Frequency in First Scenario	Status Transition Frequency in Second Scenario	Percentage Change (%)	Overall Link Connectivity Increase/Decrease
TF Parameter	Fv1 = 10	Fv1 = Fv2 = 10	25%	Decrease
	Fv3 = 8	Fv3 = Fv4 = 8	40%	Decrease
	Fv5 = 6	Fv5 = Fv6 = 6	55%	Decrease
	Fv7 = 4	Fv7 = Fv8 = 4	70%	Decrease

**Table 5:** Effect of residual energy on link connectivity.

Parameter	Initial Residual Energies in First Scenario	Initial Residual Energies in Second Scenario	Change in Initial Residual Energies (%)	Final Residual Energies in First Scenario	Final Residual Energies in Second Scenario	Change in Final Residual Energies (%)	Overall Link Connectivity Increase/Decrease
Residual Energy	R_E1_ = 0.99	R_E1_ = 0.99, R_E2_ = 0.96	4%	R_E1_ = 0.72	R_E1_ = 0.72, R_E2_ = 0.65	35%	Decrease
	R_E3_ = 0.94	R_E3_ = 0.94, R_E4_ = 0.99	1%	R_E3_ = 0.60	R_E3_ = 0.60, R_E4_ = 0.74	26%	Decrease
	R_E5_ = 0.84	R_E5_ = 0.84, R_E6_ = 0.94	6%	R_E5_ = 0.60	R_E5_ = 0.60, R_E6_ = 0.78	22%	Decrease
	R_E7_ = 0.94	R_E7_ = 0.94, R_E8_ = 0.74	26%	R_E7_ = 0.78	R_E7_ = 0.78, R_E8_ = 0.48	52%	Decrease
